# Peptide-mimetics derived from leucyl-tRNA synthetase are potential agents for the therapy of mt-tRNA related diseases

**DOI:** 10.3389/fphar.2025.1607343

**Published:** 2025-07-28

**Authors:** Annalinda Pisano, Sara Belloli, Maria Gemma Pignataro, Paolo Rainone, Silvia Valtorta, Angela Coliva, Valeria de Turris, Luciana Mosca, Patrizio Di Micco, Rosa Maria Moresco, Giulia d’Amati, Veronica Morea

**Affiliations:** ^1^ Department of Radiology, Oncology and Pathology, “Sapienza” University of Rome, Rome, Italy; ^2^ Institute of Bioimaging and Biological Complex Systems, National Research Council of Italy, Segrate, Italy; ^3^ Department of Nuclear Medicine, IRCCS San Raffaele Scientific Institute, Milan, Italy; ^4^ Center for Life Nano- & Neuro-Science, Fondazione Istituto Italiano di Tecnologia (IIT), Rome, Italy; ^5^ Department of Biochemical Sciences “A. Rossi Fanelli”, “Sapienza” University of Rome, Rome, Italy; ^6^ Institute of Molecular Biology and Pathology, National Research Council of Italy, Rome, Italy; ^7^ Department of Medicine and Surgery, University of Milano-Bicocca, Monza, Italy

**Keywords:** peptide-mimetic molecules, MELAS and MERRF cybrids, rescuing effect, plasma stability, biodistribution, PET, Cu-64 radioisotope, tolerability

## Abstract

**Introduction:**

Mitochondrial diseases caused by point mutations in mitochondrial tRNA (mt-tRNA) genes, including MELAS and MERRF syndromes, represent a significant unmet clinical need, due to the lack of effective treatments. We previously identified peptide molecules derived from human leucyl-tRNA synthetase, whose features make them attractive leads for the development of therapeutic agents against mt-tRNA point mutations-related diseases. Indeed, we demonstrated that, upon exogenous administration, these peptides penetrate human cell and mitochondrial membranes; stabilize mitochondrial tRNA structures; and rescue severe mitochondrial defects in cells bearing the point mutations m.3243A>G and m.8344A>G, responsible for MELAS and MERRF syndromes, respectively.

**Results:**

To progress towards therapeutic applications, in this work we designed three peptide-mimetic derivatives (PMTs). These are composed entirely of D-amino acids and potentially endowed with enhanced stability in human plasma and resistance to enzymatic degradation. We show that, like the parent peptide, the PMTs have mitochondrial localization and improve cell viability and oxygen consumption in human cybrid cell lines bearing the aforementioned point mutations. Additionally, as anticipated, the PMTs had significantly higher plasma stability than the parent peptide. The most promising PMT was radiolabelled with Cu-64 and used in *in vivo* biodistribution and tolerability studies. Importantly, i. v. administered PMT reached all body districts, including heart, muscle and even brain, thus revealing an intrinsic ability to cross the blood-brain barrier. Finally, PMT was safe in adult wild-type mice at dosages up to 10 mg/kg.

**Discussion:**

These findings represent a significant step towards the implementation of therapeutic strategies for mttRNA-related mitochondrial diseases.

## 1 Introduction

Mitochondrial (mt) diseases caused by mutations in transfer RNA (tRNA) genes are chronic, progressive, and ultimately fatal syndromes with a wide spectrum of phenotypic expression, affecting different organs and tissues, especially those with high energy requirement (e.g., CNS, heart and skeletal muscle) (Craven et al., 2017). Diverse approaches to counteract these disorders have been proposed in the past decades, such as the replacement of mutated mtDNA using gene therapy and stem cell therapy, the delivery of proteins affecting mtDNA expression and replication, antioxidant molecules, and small molecule libraries screening (Hashimoto et al., 2015; Nightingale et al., 2016; Hirano et al., 2018; Barcelos et al., 2020; Bottani et al., 2020; Gunnewiek et al., 2021). However, the difficulty posed by the presence of mutations in thousands of copies of mtDNA per cell has prevented the development of therapies able to effectively counteract the symptoms of these diseases, making them an unmet clinical need.

In recent years, we have developed an original approach that has proved to be effective in rescuing the defective phenotype of human cell models bearing point mutations in genes coding for mt-tRNAs (Perli et al., 2014; Perli et al., 2016; Perli et al., 2020). This approach has its roots in the observation that the overexpression of mt-tRNA interacting proteins encoded by the nucleus, such as aminoacyl-tRNA synthetases (aaRSs) or the elongation factor (EF)-Tu, rescues the defective phenotype caused by mt-tRNA mutations in cellular models like yeast or human cytoplasmic hybrids (cybrids) (Francisci et al., 2005; Montanari et al., 2008; Park et al., 2008; Rorbach et al., 2008; De Luca et al., 2009; Li and Guan, 2010; Montanari et al., 2011; Perli et al., 2014; Perli et al., 2016; Perli et al., 2020). Interestingly, we demonstrated that the rescuing effect of leucyl-tRNA synthetase (LeuRS) towards cybrids bearing the m.3243A>G mutation in *MT-TL1* gene, coding for mt-tRNA^Leu(UUR)^, resides entirely in the 67 amino acid (a.a.) long C-terminal domain (Cterm) (Perli et al., 2014) that, like EF-Tu, does not possess enzymatic activity. Analysis of the experimentally determined 3D structures of bacterial LeuRS in complex with tRNALeu (Tukalo et al., 2005; Palencia et al., 2012) available from the protein data bank (PDB) (rcsb.org; Berman et al., 2000), revealed that almost all the interactions between the bacterial Cterm and tRNA were mediated by two linear peptide regions, corresponding to LeuRS β-strands β30_31 (15 a.a. long) and β32_33 (16 a.a. long), which are conserved in the human Cterm. We demonstrated that human β30_31 and β32_33 peptides exerted the same rescuing activity as the Cterm following transfection of mutant trans-mitochondrial hybrids (cybrids) with plasmids encoding either of them (Perli et al., 2016). Further, we demonstrated that, upon direct exogenous administration to mutant cybrids, both peptides penetrate cell membranes, localize at the mt level and correct the defective phenotypes of human cells bearing either the mutation m.3243A>G in *MT-TL1* gene, or the m.8344A>G mutation in *MT-TK* human gene, encoding mt-tRNA^Lys^ (Perli et al., 2020). These are the two mutations collectively responsible for the most common and severe human mt-tRNA-related diseases (about 85%) (Craven et al., 2017; Gorman et al., 2015), namely, MELAS (Mitochondrial Encephalopathy, Lactic Acidosis and Stroke-like episodes)/MIDD (Maternally Inherited Diabetes and Deafness), associated with the m.3243A>G mutation, and MERRF (Myoclonic Epilepsy with Ragged Red Fibers), associated with the m.8344A>G mutation. Finally, we demonstrated that both β30_31 and β32_33 peptides are able to interact with high affinity with both mt-tRNA^Leu(UUR)^ and mt-tRNA^Lys^ bearing the aforementioned mutations and stabilize a conformation of the mutated tRNA similar to the respective wild-type molecules (Perli et al., 2020). These results prompted us to hypothesize a chaperone-like mechanism of action of the peptides, according to which: pathogenic mutations destabilize the native structure of mt-tRNAs, which assume a conformation less suitable to interact with macromolecular partners such as aaRSs, modification enzymes, ribosome, etc., thereby causing the defective phenotype; by directly interacting with mutated mt-tRNAs, rescuing molecules would stabilize a conformation of the mutated mt-tRNA similar to those of the wild-type molecules, whose fitness to perform mt-tRNA functions has been selected by evolution.

Once *in vitro* active peptides have been identified, biodistribution and *in vivo* tolerability studies must be performed to move forward toward potential clinical applications. Since peptide bonds are susceptible to enzymatic cleavage, in the present work we designed three peptide-mimetic derivatives (PMTs) of the β32_33 peptide, which is more active than the β30_31, and assessed their ability to rescue the pathological phenotype of human cells carrying the mutation m.3243A>G or m.8344A>G. Then, we evaluated PMTs stability in human plasma, with respect to β32_33, by Liquid Chromatography coupled to Mass Spectrometry. Based on the results of these studies, the PMT endowed with the highest rescuing activity and higher plasma stability than β32_33 was selected for *in vivo* biodistribution and tolerability studies.

To assess the PMT ability to reach different organs after intravenous (i.v.) administration in mice, we used Positron Emission Tomography (PET), a molecular imaging technique able to quantitatively detect radiolabelled probes within animal models (Yao et al., 2012), as well as a reliable tool to assess therapy efficacy, in a context of increasingly personalised patient management (Ricci et al., 2020; Pruis et al., 2020). This technology exploits the use of radioactive imaging probes, designed to target different processes or markers within the body, which can be visualised according to their specific expression or accumulation in the body districts (Gambhir, 2002). To evaluate the pharmacokinetic properties of PMT, a radiolabelling procedure was set up. The Cu-64 radioisotope, whose half-life is 12.7 h, was used to track PMT *in vivo* kinetics up to 48 h after administration. *In vivo* studies demonstrated that the PMT is well tolerated and reaches all the investigated organs, including those that, because of high energy consumption, are most affected by mutations in mt-tRNA genes, namely, heart, skeletal muscle and even, although at lower levels, brain. These results indicate that the PMT can be considered a promising therapeutic candidate for MELAS, MIDD and MERRF syndromes.

Since animal models bearing mt-tRNA mutations with a clear clinical phenotype have not been developed yet, further *in vivo* investigations will be aimed at the identification of optimal administration protocols to bring the PMT to target tissues in dosages comparable to those that exert a rescuing activity in cell models, possibly by exploiting *ad hoc* delivery strategies.

## 2 Materials and methods

### 2.1 Peptide design and synthesis

The peptide-mimetic compounds were designed to have the following features: i) therapeutic activity and safety at least as high as the β32_33 peptide in cell models; ii) higher stability than the β32_33 peptide in human plasma; iii) ability to reach the target organs and tissues, and in particular heart, skeletal muscle, and central nervous system (CNS) upon *in vivo* administration. To reach these goals, we designed three peptide-mimetic compounds comprising only amino acids with a D configuration, which is rarely used by living organisms, as opposed to the naturally occurring L configuration. Therefore, neither peptide-mimetic is expected to undergo either proteolytic cleavage or other stereospecific enzyme-catalysed transformation. Specifically, the three peptides are: the PMT, which comprises the D-enantiomers of the L-amino acids present in the β32_33 peptide, in the same order; and the PMT-8a and PMT-8b, which are fragments of the PMT, comprising the D-amino acids 1-8 and 5–12 of the PMT, respectively.

All constructs were synthesized with purity >85% by Pepscan, now part of the Biosynth Group (Biosynth B.V., Zuidersluisweg 2, 8243 RC Lelystad, The Netherlands). The compounds used for the study are listed in Table 1.

**TABLE 1:** Peptides and constructs studied in this work. Fluorogenic constructs are indicated by “-C” at the end of their names. En, enantiomer. Link, cross-linker between the C-terminal a.a. of the peptide and the fluorophore. Mal, maleimide. Fluo, fluorophore. MW, molecular weight.

Name	En	A.a. sequence	Link	Fluo	MW (Da)
βp (β32_33)	L	KKSFLSPRTALINFLV			1833.4
PMT	D	KKSFLSPRTALINFLV			1833.4
PMT-8a	D	KKSFLSPR			961.3
PMT-8b	D	LSPRTALI			869.2
βp-C	L	KKSFLSPRTALINFLV	Mal	Cy5	2543.2
PMT-C	D	KKSFLSPRTALINFLV	Mal	Cy5	2542.3
PMT-8a-C	D	KKSFLSPR	Mal	Cy5	1670.2
PMT-8b-C	D	LSPRTALI	Mal	Cy5	1578.2
^64^Cu-PMT	D	KKSFLSPRTALINFLV	Mal	NOTA	2426.9

### 2.2 3D structure analysis and molecular modelling

The experimentally determined 3D structures of bacterial LeuRS in complex with tRNA^Leu^ were downloaded from the Protein Data Bank (PDB) (Berman et al., 2000). Their PDB identifier (ID), Resolution and source are the following: PDB ID: 2BTE, 2.90 Å, *T. thermophilus* (Tukalo et al., 2005) and PDB ID: 4AQ7, 2.5 Å, *E. coli* (Palencia et al., 2012).

The sequences of human mt-LeuRS and mt-tRNA^Leu(UUR)^ were downloaded from the UniProt database (uniprot.org; ID: Q15031) (UniProt Consortium, 2005) and the National Center for Biotechnology Information (NCBI) database of gene sequences (https://www.ncbi.nlm.nih.gov/; ID: NC_012920.1:3230-3304), respectively.

Molecular docking of the 3D models of human LeuRS and mt-tRNA^Leu(UUR)^ was performed using by the freely available LZerD (https://lzerd.kiharalab.org/; Christoffer et al., 2021) and AlphaFold3 (https://alphafoldserver.com/welcome; Abramson et al., 2024) web servers.

Swiss-PDBViewer (http://www.expasy.org/spdbv/; Guex and Peitsch, 1997) was used for 3D structures visualization, analysis and modelling and FACE2FACE (https://face2face.ibpm.cnr.it/; Di Micco et al., unpublished) to calculate intermolecular contacts and generate 2D contact maps.

### 2.3 Cell lines

Previously established osteosarcoma-derived (143B.TK-) cybrid cell lines from patients, which bear either the m.3243A>G mutation in mt-tRNA^Leu(UUR)^ or the m.8344A>G mutation in mt-tRNA^Lys^ and controls (Perli et al., 2014; Perli et al., 2016; Perli et al., 2020) were used. The pathological mt-tRNA^Leu(UUR)^ mutant had a mutation load >98%. The mt-tRNA^Lys^ mutant had a mutation load of either ∼80% (H-8344) or ∼30% (L-8344). The H-8344 high mutation load mutant was pathological, whereas the L-8344 low mutation load mutant did not show any detectable phenotype and was used as control (Perli et al., 2020).

### 2.4 Cell culture

Cybrid cells were cultured in Dulbecco’s modified Eagle’s medium (DMEM), supplemented with 4.5 g/L D-glucose, 10% foetal bovine serum (FBS), 2 mM L-glutamine, 50 μg/ML uridine, 100 U/mL penicillin, and 100 mg/mL streptomycin (referred to as glucose medium) in a humidified atmosphere of 95% air and 5% CO_2_ at 37°C. For cell viability experiments, cells were grown either in glucose medium or in glucose-free DMEM, supplemented with 5 mM galactose, 110 mg/mL sodium pyruvate, and 10% FBS (referred to as galactose medium). The reason for using the latter medium is that a pathological phenotype can be appreciated in cells growing on galactose, which forces cells to rely on mitochondrial respiration, but not in cells growing on glucose.

### 2.5 Confocal fluorescence microscopy

Constructs made of compounds listed in Table 1, comprising D-peptides linked to the Cy5 fluorophore via maleimide cross-linker, were administered to sub-confluent cybrid cell cultures at 0.25 µM in glucose medium. About 24 h after treatment with each construct, cells were incubated with 200 nM Mitotracker Red FM (LifeTechnologies Italia, Monza, Italy) for 30 min at 37°C.

Subsequently, cells were visualized by confocal microscopy. Images of 800 × 800 px (at 88 nm/px) were acquired at the Olympus iX83 FluoView1200 laser scanning confocal microscope using a 60 × NA1,35 oil objective (Olympus Italia SRL Milano, Italy), zoom 3×, 559 nm, and 635 nm lasers and filter setting for Mitotracker Red and Cy5. The fluorescence images were analysed with the ImageJ software (Rasband, 2018) to determine the Pearson’s correlation coefficient.

For a subset of experiments, we evaluated the mt localization of constructs up to 72 h after treatment. PMT and PMT fragments were administered to sub-confluent cybrids at a concentration of 0.25 µM in the glucose medium. At 24-, 48- and 72-h after treatment, cells were incubated with Mitotracker Red and visualized by confocal microscopy. After each confocal imaging section, a fresh glucose medium was added to the cells, which were maintained under standard conditions (a humidified atmosphere of 95% air and 5% CO_2_ at 37°C).

### 2.6 Cell viability

To assess growth capability, cells were harvested and seeded at 30 × 10^4^ in 60 mm dishes in glucose medium for 24 h with the addition of one of the compounds. Cells were switched in glucose or galactose and, after 24 h, cell viability was measured by the Trypan blue dye exclusion assay. Cells were harvested with 0.25% trypsin and 0.2% EDTA, washed, suspended in PBS in the presence of Trypan blue solution (Sigma–Aldrich) at 1:1 ratio and counted using a hemocytometer. The number of viable cells in the galactose medium was expressed as a percentage of the number of cells in glucose medium.

### 2.7 Respirometry assay

Oxygen consumption rate (OCR) of cybrids was evaluated with Clark type oxygen electrode (Hansatech Instruments, Norfolk, United Kingdom). After cybrid incubation with compounds at 5 µM concentration, both control and mutant cybrids were maintained in glucose medium for 36 h, then OCR was measured in intact cells (3 × 10^6^) in 1 mL DMEM lacking glucose supplemented with 10% sodium pyruvate.

### 2.8 Cell and mitochondrial toxicity

Mitochondrial toxicity exerted by PMT was measured using the Mitochondrial ToxGlo™ Assay (Promega Italia S. r.l., Milano, Italy) according to the manufacturer’s protocol. Cybrids were plated on a 96-well plate and treated with different PMT concentrations (0.5, 2, 5, 10, 20, 30, 40 and 50 µM). Twenty-four hours after treatment, control cells (both wild-type and mutated) were incubated with either 400 μg/mL digitonin (a cytotoxic agent) or 50 µM sodium azide (a mitotoxic agent) for 3 h, as positive controls for cyto- or mito-toxicity, respectively. Subsequently, cells were incubated with specific reagents and fluorescence or luminescence were measured with a GloMax Multi + Luminometer (Promega Italia Srl., Milano, Italy).

### 2.9 Plasma stability

To assess whether the β32_33 peptide, PMT, PMT-8a and PMT-8b peptide-mimetics are stable in blood, we set up a chromatographic assay able to evaluate compound concentration after incubation in human plasma.

Four different samples from healthy volunteers were used. Blood was drawn by venepuncture in vacutainer containing EDTA as an anticoagulant. Plasma was separated by centrifugation and immediately used for the experiments. Each compound was dissolved in 500 μL plasma at a final concentration of 0.2 mM and split into two aliquots, one of which was immediately analysed to assess the basal compound level (T_0_); the other was incubated at 37°C under gentle shaking, and the amount of each compound was measured at different time points (i.e., 1.5, 3, 6 and 72 h).

To perform chromatographic analyses, the samples were treated with 3 volumes of acetonitrile containing 1% formic acid and then extracted by using the Ostro™ pass-through sample preparation system to remove proteins and phospholipids. Samples were dried under vacuum, resuspended in 100 μL of 0.1% formic acid containing 5% acetonitrile, and then injected directly onto the chromatographic column.

Chromatographic analyses were performed on a Water Acquity H-Class UPLC system (Waters, Milford, MA, United States), including a quaternary solvent manager (QSM), a sample manager with a flow-through needle system (FTN), a photodiode array detector (PDA) and a single-quadruple mass detector with electrospray ionization source (ACQUITY QDa). Analyses were performed on a reverse phase C18 column (75 mm × 3.2 mm i. d., 2.5 μm particle size). The mobile phase was solvent A, 0.1% formic acid in water, and solvent B, 0.1% formic acid in acetonitrile. The flow rate was 0.5 mL/min, the column temperature was set at 25°C and the elution was performed by linearly increasing the concentration of solvent B up to 70% in 7 min. Mass spectrometric detection was performed in the positive electrospray ionization mode, using nitrogen as the nebulizer gas. Analyses were performed in the Total Ion Current (TIC) mode with a mass range of 100–1200 m/z. The capillary voltage was 0.8 kV, cone voltage 8 V, ion source temperature 120°C and probe temperature 600°C. Quantification was performed by Selected Ion Recording (SIR): at m/z = 917.88, corresponding to the [M+2H]^2+^ ion obtained from either *β*32_33 peptide or PMT; at m/z = 961.42, corresponding to the [M + H] ^+^ obtained from PMT-8a; and at m/z = 869.48, corresponding to the [M + H]^+^ ion obtained from PMT-8b.

### 2.10 Radiolabelling

The radiolabelling protocol was optimized by investigating different conditions for pH, peptide amounts, temperature, and time of reaction. During the setup, the absence of unreacted copper by SPE tC18 cartridges was ascertained, therefore a subsequent purification step was not required. The final protocol was as follows: 370 MBq of [^64^Cu]CuCl_2_ in HCl 0.1M were added to 240–250 μg of PMT-NOTA peptide in buffer sodium acetate 0.4 M, pH 7. The reaction mixture was incubated at 70°C for 30 min. The mixture was allowed to cool down to room temperature before quality control. The radiochemical purity of ^64^Cu-PMT was assessed by reversed phase HPLC on C18 column by applying a gradient elution with water/acetonitrile added with 0.1% TFA.

### 2.11 Animals and study design

Two-month-old male C57BL/6J mice were purchased from ENVIGO RMS S. r.l. (San Pietro al Natisone, Italy). All animals were housed in the San Raffaele Research Institute (Milan) animal facility under constant temperature and humidity, with a 12-h light/dark cycle and access to food and water *ad libitum* and environmental enrichment. Experimental procedures involving the use of animals complied with the EU Directive 2010/63/EU for animal experiments and have been approved by the Ethical Committees of the San Raffaele Research Institute (Milan) and the Italian Ministry of Health (license n. 717/2021-PR). The study was conducted in compliance with the ARRIVE 2.0 guidelines.

Sixty adult C57BL/6 male mice were initially included in the study. Twenty animals were dedicated to *in vivo*/*ex vivo* biodistribution of the radiolabelled PMT ^64^Cu-PMT. The evaluation was carried out at four time points: 1, 3, 24 and 48 h. Forty animals were dedicated to tolerability studies with increasing PMT concentration: 1, 5, 10 and 50 mg/kg PMT or saline, i. v. (n = 10 per group). The highest dose was evaluated only in two animals, since they died immediately after the administration. Therefore, only three groups of ten animals were dedicated to the tolerability test, and fifty C57BL/6 male mice were employed for the current study in total. Necropsy of the two animals injected with 50 mg/kg PMT did not highlight any obvious macroscopic effect, and no further molecular investigations were done. Due to the rapidity of death, and the hydrophobic character of the C-terminal region of the PMT (see the Discussion section), it is possible that PMT aggregates obstructed one or more small vessels, causing micro-infarctions in vital organs (e.g., lung, heart, brain).

### 2.12 *In vivo* PET-CT and *ex vivo* distribution of radiolabelled ^64^Cu-PMT in healthy animals

PET-CT imaging was performed using β-Cube^®^ and γ-Cube^®^ (Molecubes, Gent, NE), respectively, equipped with the mouse Hotel insert hosting up to four mice simultaneously. A group of 5 animals were injected into the caudal vein with the radiopharmaceutical ^64^Cu-PMT (mean injected radioactivity of 3.62 ± 1.03 MBq, corresponding to mean injected mass of 4.15 ± 0.53 µg) and subjected to PET-CT acquisition at 1, 3, 24 and 48 h after injection. For each time point, animals were anesthetized by isoflurane (2% in medical air) and placed prone on the CT scanner bed for CT acquisition (2 min after scout). Thereafter, the bed with the immobilized animals was moved in the PET scanner for the acquisition (30 min). Temperature was maintained constant, and respiratory and cardiac rates were monitored. At the end of the last acquisition (48 h), animals were sacrificed by decapitation, followed by immediate blood recovery by cardiac puncture. An aliquot of blood was centrifuged to obtain the plasma. The following tissues were also dissected: liver, heart, lungs, spleen, intestine, stomach, kidney, muscle, and brain. All samples were placed in pre-weighed tubes for gamma-counter counting. After decay, the tubes were weighted again for tissue net weight determination to calculate the percentage of injected dose of radioactive compound per gram of tissue (%ID/g).

Fifteen additional animals were injected with the same dose of radioactive peptide as the other five and sacrificed at 1, 3, and 24 h (n = 5 per time point) for gamma-counting of tissues, to compare *ex vivo* biodistribution with *in vivo* PET/CT results. Animals were anesthetized with isoflurane and sacrificed by cardiac puncture. An aliquot of blood was centrifuged to obtain the plasma. Liver, heart, lungs, spleen, intestine, stomach, kidney, muscle, and brain were also dissected and counted to gamma-counter as described above.

PET and CT data collected for each animal and at each time point were reconstructed and automatically co-registered. Images were then corrected for isotope decay and analysed using the software PMOD 4.1; areas of interest were quantified using Volumes of Interest (VOIs) analysis. VOIs were manually drawn on transaxial sections within liver, heart wall, kidney, muscle, cerebrum, and cerebellum, and radiolabelled PMT signal was automatically extracted. Data were expressed as SUV_mean_ (Standardized Uptake Value), the index usually adopted to express PET data, which is corrected for animals’ body mass.

### 2.13 Tolerability in animals

Adult C57BL/6 male mice were used to determine the general tolerability of i. v. administered PMT and identify the Maximum Tolerated Dose (MTD). To this purpose, animals were divided into four groups (n = 10 each) and injected in a tail vein with either saline (controls) or PMT freshly dissolved in saline at three scalar doses (1, 5 or 10 mg/kg). Each group was randomly divided in two subgroups (n = 5 each). Blood ocular withdrawal was performed at 1 h and 48 h post-injection for each subgroup, respectively. During this procedure, animals were anaesthetized with isoflurane (2% in medical air) and monitored until awakening.

The effect of PMT administration was evaluated every 2 days by observation of the animals and application of the following criteria and scores (see Supplementary Table S1): body weight loss (score: 1–5); coat conditions (score: 1–3); dehydration (score: 1–3); respiration (score: 1–3); spontaneous behaviour (score: 0–5); reaction to handling (score: 0–3) (IACUC of the San Raffaele Research Institute and FELASA Glossary 2015 (Fentener van Vlissingen et al., 2015).

The observation was carried out for 2 weeks post peptide injection. At the end, all the animals underwent a grid-hanging test to measure muscular strength performances after PMT or saline treatment (Gayi et al., 2018).

After a period of habituation to the metallic grid, mice were placed individually on top of a metallic grid (40 × 40 cm, 1.3 cm square mesh) suspended 50 cm above an empty cage. The grid was turned upside down and latency to fall was measured up to a fixed limit of 600 s. Each mouse performed three trials and the longest holding time (s) among them was multiplied by the animal’s body weight (g) to calculate Holding impulse (g*s).

After the test, mice were sacrificed under general anaesthesia (4% isoflurane in medical air) by cardiac puncture to withdraw blood, brain, heart, liver, kidney, spleen, and lymph nodes, which were collected into tubes with buffered formalin and stored at +4°C.

Samples of blood collected at 1 h, 48 h and 15 days were centrifuged to obtain plasma that was transferred to cryovials and stored at −80°C.

### 2.14 Morphological analysis of selected organs

At the end of the tolerability test, tissues collected from animals injected with saline (n = 5) or treated with 1, 5 and 10 mg/kg of PMT (n = 10), were formalin-fixed and paraffin-embedded. Five µm-thick sections were obtained from the paraffin blocks and stained with hematoxylin and eosin for histologic examination.

### 2.15 Statistical analysis

#### 2.15.1 Cell experiments

All data are expressed as mean ± SEM. Data were analysed by standard ANOVA procedures followed by multiple pair-wise comparisons adjusted with Bonferroni corrections. Significance was considered at *p* < 0.05. Numerical estimates were obtained with Graphpad Prism 7 version (Graphpad Inc. San Diego, CA, United States).

#### 2.15.2 Animal experiments

Data on PMT tolerability in animals (*i.e.*, body mass monitoring and grip-hanging test) are expressed as mean ± SD and analysed using one-way ANOVA corrected for multiple comparison Turkey test.

## 3 Results

### 3.1 PMT, PMT-8a and PMT-8b penetrate cell membranes and co-localize with mitochondria

**FIGURE 1:**
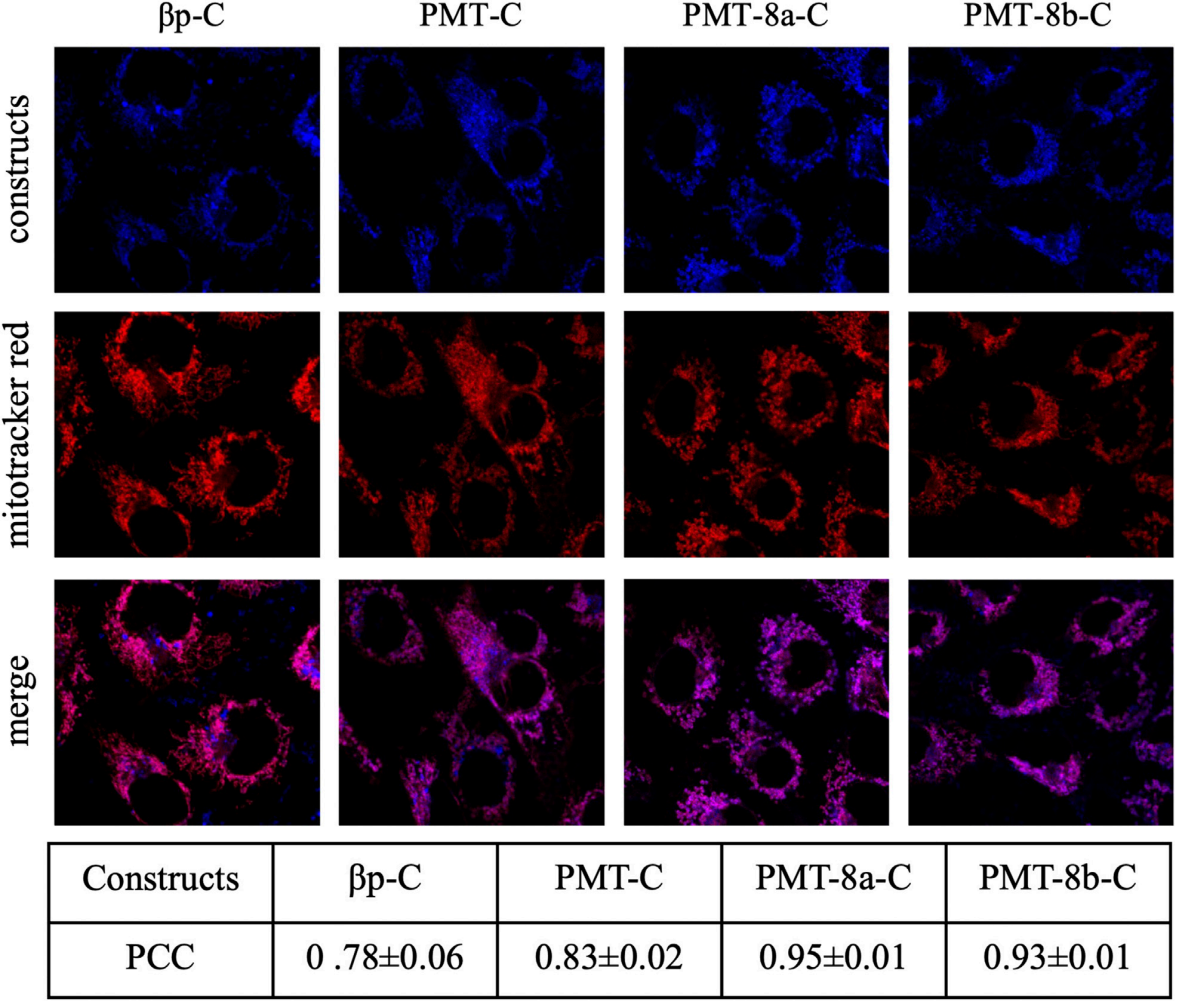
Upon exogenous administration to mutant cells, PMT and PMT fragments (PMT-8a and PMT-8b) penetrate cell membranes and colocalize with mitochondria. Cybrids were incubated with 0.25 μM of constructs and imaged after 24 h. Half an hour before imaging, cells were stained with Mitotracker Red. PCC: Pearson’s Correlation Coefficient (mean ± SEM of at least five images). Names of all constructs are followed by “-C” to indicate that they are covalently linked to the Cy5 fluorescent dye.

Confocal microscopy experiments showed that, upon exogenous administration to mutant cells, the fluorescent signal of PMT and PMT fragments (PMT-8a and PMT-8b) was detectable within cybrids and overlapped with the mt reticulum, indicating mt localization, as demonstrated by Pearson’s correlation coefficients (PCC values: 0.78 ± 0.06, 0.83 ± 0.02, 0.95 ± 0.01 and 0.93 ± 0.01 for βp, PMT, PMT-8a and PMT-8b respectively; Figure 1). The fluorescence signal remained constant over time; in fact, mt localization did not change up to 72 h, as indicated by Pearson’s correlation coefficients (Supplementary Figure S1).

### 3.2 PMT ameliorates both viability and oxygen consumption of m.3243A>G mt-tRNALeu(UUR) and m.8344A>G mt-tRNA^Lys^ mutant cybrids

**FIGURE 2:**
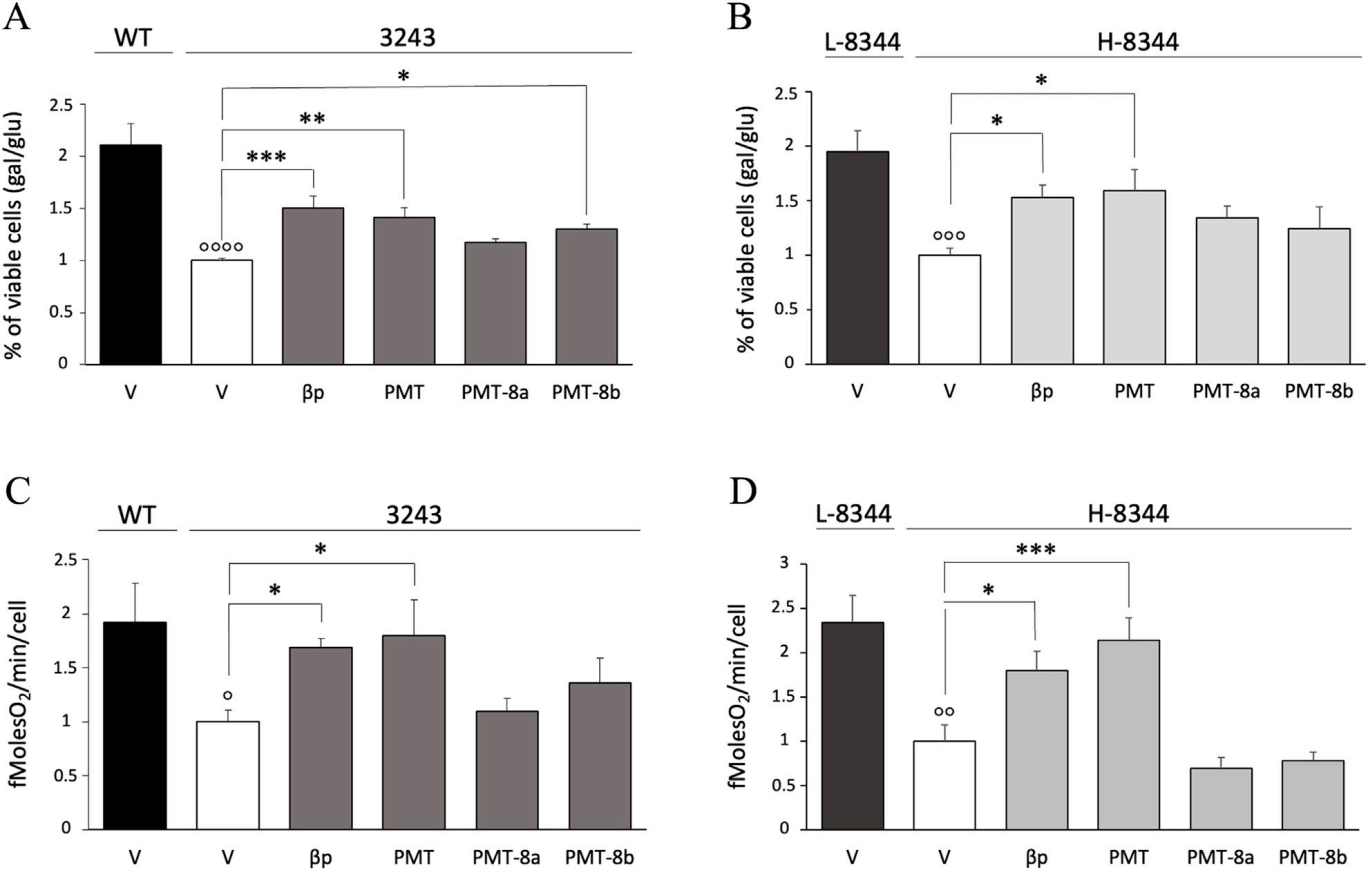
Following exogenous administration, PMT significantly improves cell viability and mt respiration of mutant cells. **(A,B)** Viability of both treated and vehicle 3243 **(A)** and H-8344 **(B)** cybrids, evaluated after 24 h incubation in galactose medium. The number of viable cells in galactose medium is normalized to the number of viable cells in glucose at the same time point. Data are compared with the value of vehicle mutant cells. Mean ± SEM of at least three independent experiments is shown. **(C,D)** Rate of oxygen consumption of 3243 **(C)** and H-8344 **(D)** cybrids, evaluated after 36 h of treatment. Compounds were administered at 5 µM concentration. Data are shown compared with the value of the vehicle mutant cells. Means ± SEM of at least three independent experiments are shown. V: vehicle; WT: wild type; 3243: m.3243A>G mutant cells. L-8344 and H-8344: low (control cells) and high (mutant cells) m.8344A>G mutation load p < 0.05,  p < 0.01,   p < 0.001    p < 0.0001 for mutant vs. control cells; *p < 0.05, **p < 0.01, ***p < 0.001 for cells incubated with compounds vs. vehicle only.

To evaluate the effect of PMT and PMT fragments on viability, cybrids were grown in glucose-free medium supplemented with galactose (galactose medium), a condition that both forces cells to rely on the mt respiratory chain for ATP synthesis and causes a significant growth reduction in the presence of mutations. Administration of 5 µM PMT induced a significant improvement of cell viability in m.3243A>G and m.8344A>G mutant cybrids, as compared with non-treated mutant cells (Figures 2A,B). PMT rescuing activity was comparable to that of the β32_33 peptide and higher than that of either PMT-8a or PMT-8b at the same concentrations as PMT. PMT-8b significantly improved cell viability only in m.3243A>G mutant (Figure 2A).

To investigate whether increased cell viability was related to improved mt bioenergetics, we analysed the respiratory capability of mutant and control cells by using the Clark type electrode. PMT determined a significant increase in oxygen consumption rate in both the m.3243A>G and m.8344A>G pathological mutants (Figures 2C,D). This increase was higher than that determined by either PMT-8a or PMT-8b. The same treatment had no effect on the viability and oxygen consumption of control cells (Supplementary Figure S2A–D).

Given that PMT ameliorated cell viability and increased oxygen consumption to a higher extent with respect to either PMT-8a or PMT-8b, we performed subsequent experiments on PMT alone.

**FIGURE 3:**
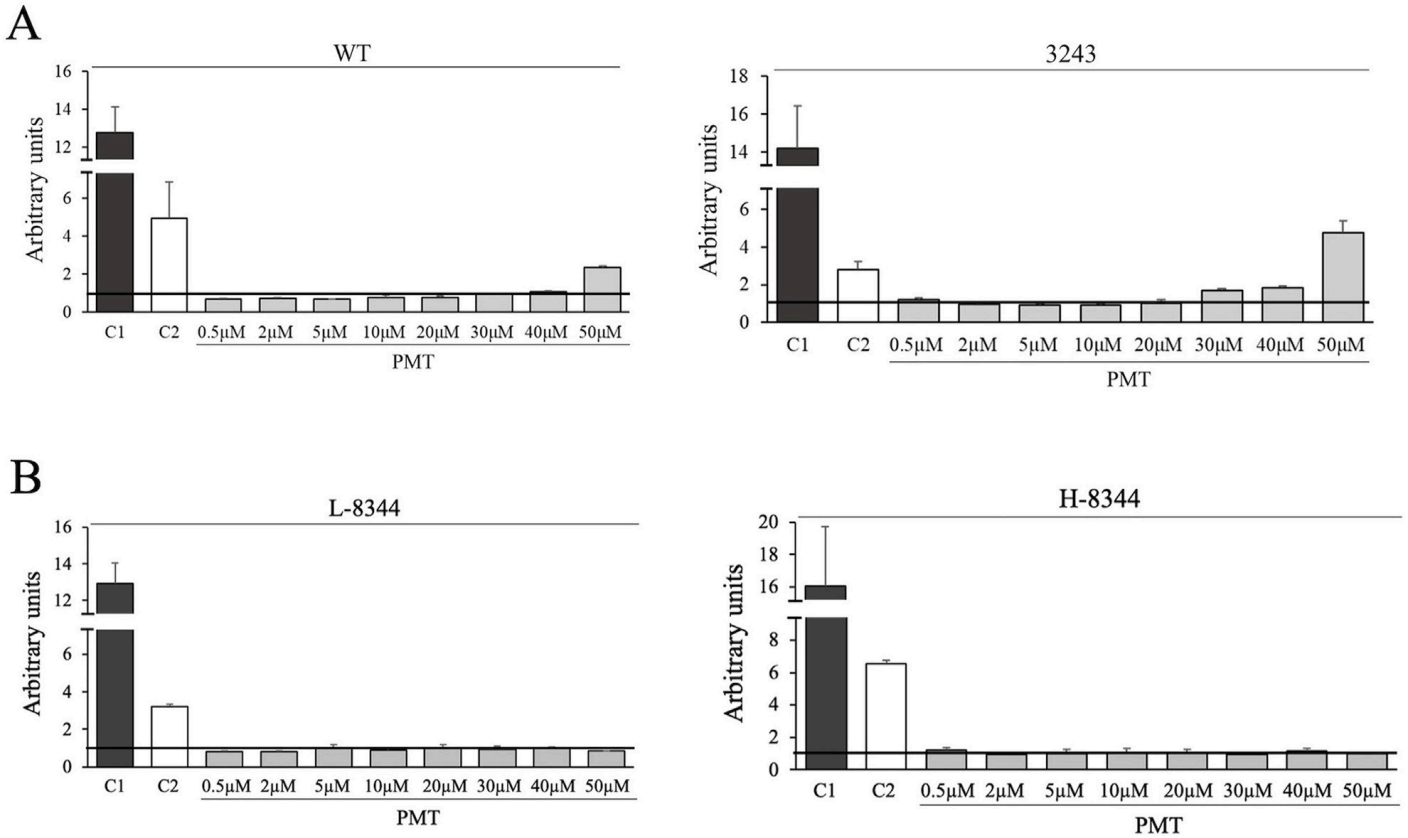
Exogenously administered PMT is neither cyto- nor mito-toxic up to 20 µM in m.3243A>G mutant cybrids and at least 50 µM in m.8344A>G mutant cybrids. Images show the effect of increasing PMT concentrations on healthy and mutant cells, compared with the effect of a cytotoxic (C1) and a mitotoxic (C2) agent, evaluated using the Mitochondrial ToxGlo™ Assay. C1: digitonin (cytotoxic reagent); C2: sodium azide (mitotoxic agent). **(A)** WT: wild type; 3243: m.3243A>G mutant cells. **(B)** L-8344 and H-8344: low (control cells) and high (mutant cells) m.8344A>G mutation load. Data are the mean ± SEM of two independent experiments.

To evaluate the toxicity of increasing concentrations of exogenously administered PMT, we performed the Mitochondrial ToxGlo™ Assay. PMT resulted to be neither cytotoxic nor mitotoxic up to: 20 µM in m.3243A>G mutant cybrids; at least 50 µM in m.8344A>G mutant cybrids; and at least 40 µM in healthy control cells (Figures 3A,B).

### 3.3 PMT precursor homologues interact with tRNALeu exclusively through amino acid side chains in experimentally determined 3D structures and molecular models

We investigated the molecular reasons why the PMT has essentially the same therapeutic activity as the β32_33 peptide in cell models, in spite of their opposite stereochemistry. To this end, we analysed the interactions between LeuRS and tRNA^Leu^ in the experimentally determined structures of the complexes from *T. thermophilus* and *E. coli* available from the PDB (see Table 2). This analysis indicated that, in both experimental structures, LeuRS residues present in the β32_33 peptide regions interact with the tRNA^Leu^ only through their side-chain atoms.

**TABLE 2:** Percentage and (number of main-chain contacts/number of total contacts) between different regions of bacterial LeuRS and tRNA^Leu^ in experimentally determined 3D structures and between different regions human mt-LeuRS and tRNA^Leu(UUR)^ in the molecular model of the complex. Tt: *T. thermophilus*; Ec: *E. coli*; Hs: *Homo sapiens*. *T. thermophilus* and *E. coli* numbering is as in the 3D structures with PDB ID: 2BTE (Tukalo et al., 2005) and 4AQ7 (Palencia et al., 2012), respectively; *H. sapiens* numbering is according to the mt-LeuRS UniProt sequence with ID Q15031. The sequence alignment of *T. thermophilus*, *E. coli* and *H. sapiens* Cterm domains is reported in (Perli et al., 2016).

Source	Region name	a.a. Nb	Length	Polar	Non-polar	Other	Total
Tt (PDB ID: 2BTE)	β30_31	819–833	15	25% (2/8)	33% (2/6)	21% (4/19)	18% (6/33)
	β32_33	862–876	15	0% (0/2)	0% (0/7)	0% (0/18)	0% (0/27)
	Cterm	815–878	63	20% (2/10)	15% (2/13)	11% (4/37)	13% (8/60)
	LeuRS-ΔCterm	1–814	814	17% (7/41)	21% (6/28)	16% (14/87)	17% (27/156)
Ec (PDB ID: 4AQ7)	β30_31	802–816	15	0% (0/8)	0% (0/3)	0% (0/13)	0% (0/24)
	β32_33	845–859	15	0% (0/10)	0% (0/9)	0% (0/25)	0% (0/44)
	Cterm	798–860	62	0% (0/18)	0% (0/12)	0% (0/38)	0% (0/68)
	LeuRS-ΔCterm	1–797	797	51% (42/83)	13% (3/34)	46% (84/184)	43% (129/301)
Hs (AF3 model)	β30_31	840–854	15	0% (0/7)	0% (0/6)	8% (1/12)	4% (1/25)
	β32_33	886–901	16	0% (0/19)	0% (0/29)	4% (2/46)	2% (2/94)
	Cterm	837–903	67	0% (0/26)	0% (0/35)	9% (3/58)	3% (3/119)
	LeuRS-ΔCterm	1–836	836	15% (6/41)	43% (3/7)	21% (13/63)	20% (22/111)

We also analysed the molecular models of human mt-LeuRS and mt-tRNA^Leu(UUR)^ built by LZerD and AlphaFold3 (AF3) servers, two publicly available resources that provided accurate predictions of macromolecular assemblies in the 16th Community Wide Experiment on the Critical Assessment of Techniques for Protein Structure Prediction (CASP16: https://predictioncenter.org/casp16/). In the AF3 and LZerD models the two molecules are and are not placed in structurally homologous positions with respect to that observed in bacterial structures, respectively; therefore, only the AF3 best model was analysed (Supplementary Figure S3). The estimate of overall model accuracy provided by AF3, and expressed as predicted local distance difference test (pLDDT) values (Jumper et al., 2021), is very high (pLDDT >90) or high (70 > pLDDT ≥90) for most of the protein structure (49% and 39% of the atoms, respectively) and high for 81% of the tRNA structure, the remaining regions of which (18%) have low (50 > pLDDT ≥70) accuracy. In this model, the number of main chain contacts between the β32_33 region and the tRNA is very low (i.e., 2/94 = 2% of total contacts) (Table 2; Supplementary Figure S3).

### 3.4 PMT has higher stability than the β32_33 peptide in human plasma

**FIGURE 4:**
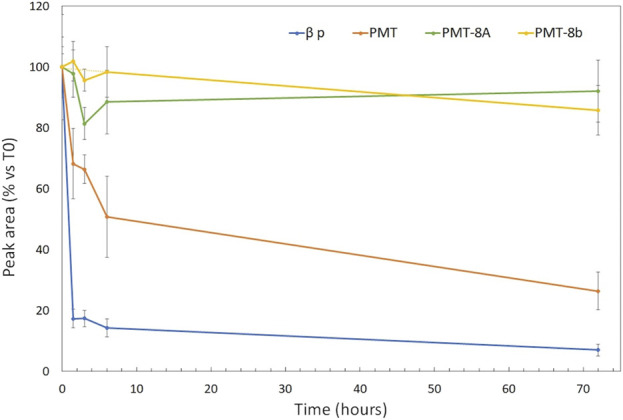
The signals of PMT-8a and PMT-8b undergo slower decrease than those of PMT and of the *β*32_33 peptide in human plasma. Time course decay of *β*32_33, PMT, PMT-8a and PMT-8b incubated up to 72 h with human plasma from four healthy subjects (blue, orange, green and yellow line, respectively). The X-axis shows the time (in hours) at which the sample is analysed. The Y-axis indicates, for each of the four compounds, the percentage of signal intensity peak area at the indicated times with respect to that at time 0.

Following incubation with human plasma, the amount of *β*32_33 peptide decreased by 90% after 1.5 h and was almost undetectable after 72 h (Figure 4). PMT had significantly higher plasma stability than *β*32_33 peptide at all time points, although the amount of detectable compound after 72 h was reduced to about 25%. Conversely, after 72 h the amount of detectable PMT-8a and PMT-8b were >90% and >85%, respectively.

### 3.5 PMT can be radiolabelled with Cu-64 resulting in a stable compound

Radiochemical purity of the ^64^Cu-PMT construct was above 99%. Specific activity, considering labelled and unlabelled peptide, was about 1.4 GBq/mg at the End of Synthesis (EOS). The radiochromatogram of ^64^Cu-PMT quality control has been inserted in the supplementary information (Supplementary Figure S4). The final radiolabelled peptide was stable in saline solution at room temperature, maintaining a radiochemical purity of 93.5% after 24 h (Supplementary Figure S4B). Data were not acquired for longer time points.

### 3.6 *Ex vivo* and *in vivo* distribution of radiolabelled peptide ^64^Cu-PMT in healthy mice

**FIGURE 5:**
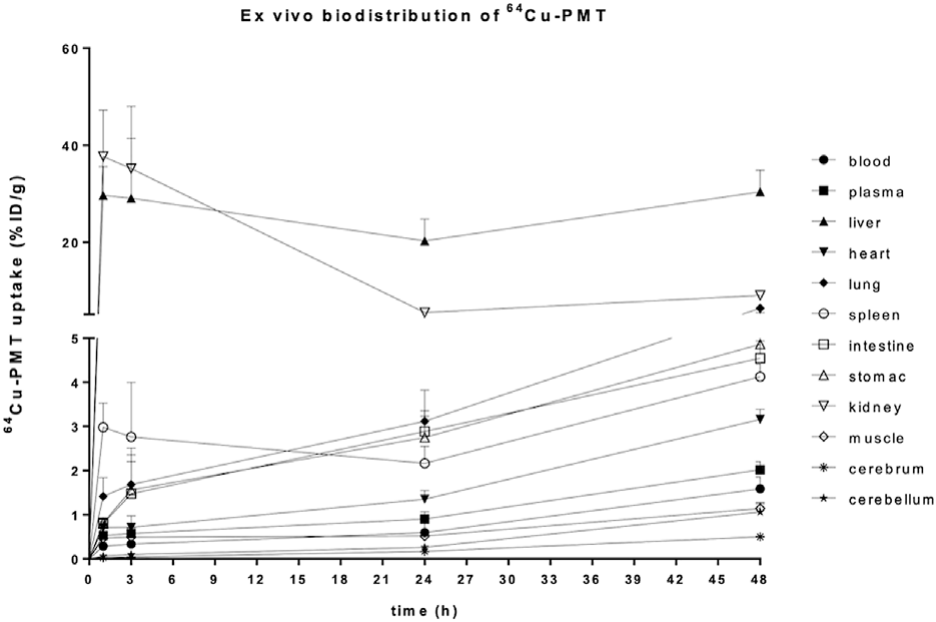
*Postmortem* biodistribution of ^64^Cu-PMT in brain and peripheral tissues of WT mice. Data are expressed as %ID/g and are mean ± SD of 5 animals per time point.

**FIGURE 6:**
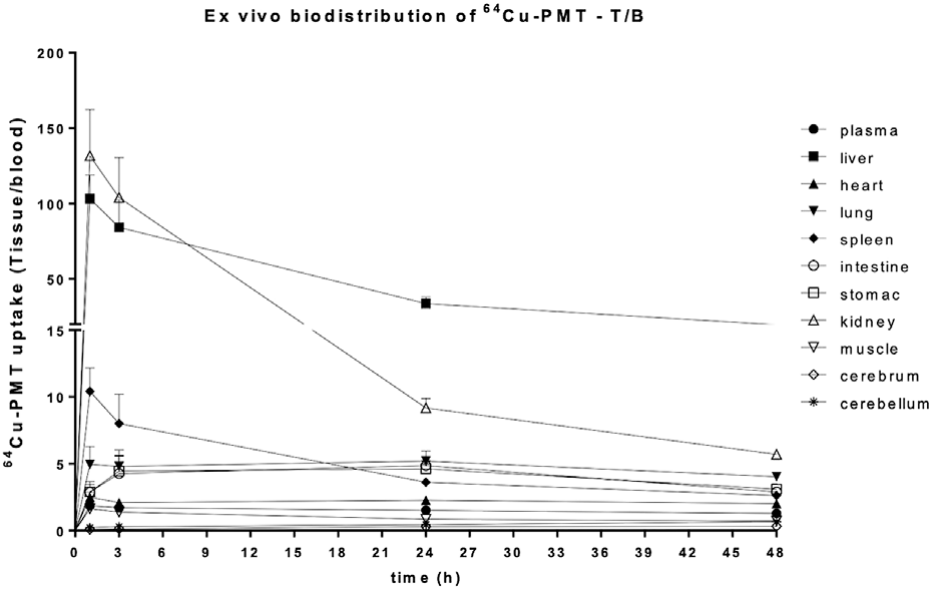
*Postmortem* biodistribution of ^64^Cu-PMT in brain and peripheral tissues of WT mice. Data are expressed as tissue to blood ratio and are mean ± SD of 5 animals per time point.

The *ex vivo* biodistribution of the radiolabelled ^64^Cu-PMT construct, expressed as %ID/g, showed an increase of radioactivity concentration in blood, plasma, brain areas and all the peripheral organs over time. The only exception was represented by the kidneys, where values peaked at 1 h, dramatically dropped at 24 h and slightly increased at 48 h post-injection in healthy mice (Figure 5; Table 3). Due to the increase in radioactivity uptake in blood over time, we also expressed data as tissue-to-blood ratios of radioactivity concentration. These data showed a decrease over time in liver, kidney and spleen, the organs devoted to the elimination of labelled PMT, while it remained stable in the other districts (Figure 6; Table 4).

**TABLE 3:** Uptake of ^64^Cu-PMT in brain and peripheral tissues of WT mice. Data are expressed as %ID/g and are mean ± SD of 5 animals per time point.

Tissue	1 h	3 h	24 h	48 h
Blood	0.291 ± 0.06	0.335 ± 0.08	0.595 ± 0.06	1.587 ± 0.26
Plasma	0.533 ± 0.08	0.573 ± 0.13	0.904 ± 0.16	2.020 ± 0.18
Liver	29.665 ± 5.93	29.081 ± 12.32	20.278 ± 4.46	30.406 ± 4.43
Heart	0.712 ± 0.13	0.715 ± 0.26	1.353 ± 0.20	3.161 ± 0.23
Lung	1.418 ± 0.42	1.684 ± 0.83	3.119 ± 0.70	6.353 ± 0.86
Spleen	2.982 ± 0.54	2.765 ± 1.23	2.165 ± 0.39	4.124 ± 0.29
Intestine	0.797 ± 0.12	1.475 ± 0.73	2.887 ± 0.47	4.547 ± 0.39
Stomach	0.822 ± 0.11	1.564 ± 0.79	2.753 ± 0.48	4.859 ± 0.58
Kidney	37.663 ± 9.55	35.195 ± 12.84	5.478 ± 0.79	8.996 ± 1.01
Muscle	0.471 ± 0.11	0.492 ± 0.29	0.520 ± 0.28	1.141 ± 0.11
cerebrum	0.026 ± 0.01	0.043 ± 0.02	0.168 ± 0.04	0.502 ± 0.04
cerebellum	0.062 ± 0.03	0.099 ± 0.04	0.260 ± 0.06	1.059 ± 0.22

**TABLE 4:** Uptake of ^64^Cu-PMT in brain and peripheral tissues of WT mice. Data are expressed as tissue to blood ratio and are mean ± SD of 5 animals per time point.

Tissue	1 h	3 h	24 h	48 h
Plasma	1.85 ± 0.15	1.71 ± 0.03	1.51 ± 0.17	1.29 ± 0.11
Liver	103.26 ± 15.60	84.24 ± 18.05	33.75 ± 4.30	19.51 ± 4.01
Heart	2.49 ± 0.38	2.09 ± 0.33	2.26 ± 0.14	2.02 ± 0.22
Lung	4.94 ± 1.35	4.80 ± 1.23	5.20 ± 0.75	4.02 ± 0.31
Spleen	10.42 ± 1.74	8.01 ± 2.19	3.61 ± 0.33	2.63 ± 0.31
Intestine	2.86 ± 0.79	4.27 ± 1.31	4.86 ± 0.70	2.89 ± 0.22
Stomach	2.89 ± 0.54	4.45 ± 1.15	4.62 ± 0.61	3.10 ± 0.42
Kidney	131.73 ± 30.56	104.06 ± 26.38	9.19 ± 0.71	5.71 ± 0.31
Muscle	1.63 ± 0.27	1.38 ± 0.48	0.85 ± 0.37	0.72 ± 0.05
cerebrum	0.33 ± 0.03	0.32 ± 0.02	0.35 ± 0.06	0.32 ± 0.04
cerebellum	0.55 ± 0.08	0.69 ± 0.07	0.59 ± 0.06	0.67 ± 0.14

**FIGURE 7:**
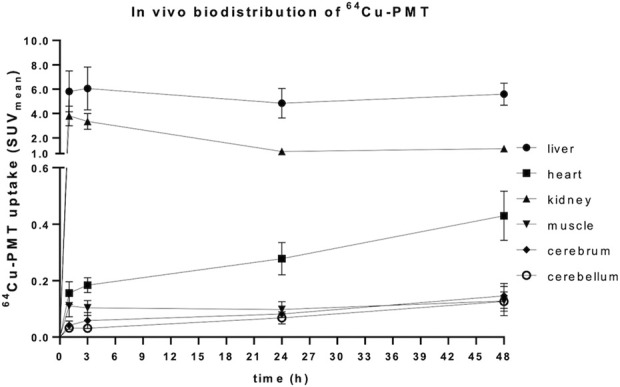
Kinetic of *in vivo* biodistribution of ^64^Cu-PMT in brain and selected peripheral tissues of WT mice. Data are expressed as SUV_mean_ and are mean ± SD of 5 animals per time point.

**FIGURE 8:**
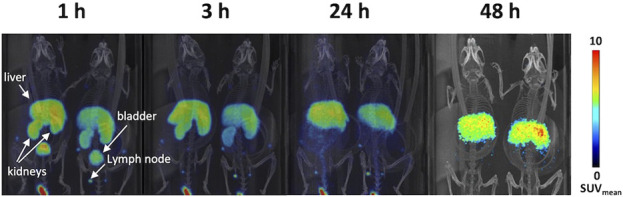
PET-CT image of representative animals acquired at the indicated time points after ^64^Cu-PMT injection. Dimensional scale bars = 1 cm.

The *in vivo* PET/CT imaging data analysis performed on peripheral and central areas are consistent with the results obtained by counting tissues by gamma counter (Figure 7; Table 5). In fact, the concentration of radiolabelled PMT reported as SUV_mean_ or %ID/g (see Figure 7; Supplementary Figure S5, respectively) increased over time in the heart wall and brain areas, whereas it remarkably decreased in the excretory organs, *i.e.*, liver and kidney, at 24 h and 48 h post injections. At variance with what is observed in *ex vivo* studies, muscle uptake remains stable. Consistent with high spleen uptake, images showed a clear PMT accumulation in lymph nodes as well (Figure 8). Interestingly, the lack of choroid plexus signal, which is the main exchange system for free copper in the brain, in the PET images indicates that free copper is present in small amounts, if any (Figure 8).

**TABLE 5:** *In vivo* uptake of ^64^Cu-PMT in brain and peripheral tissues of WT mice. Data are obtained after VOIs analysis and are expressed as SUV_mean_ ± SD of 5 animals per time point.

Tissue	1 h	3 h	24 h	48 h
Liver	5.811 ± 1.68	6.051 ± 1.77	4.845 ± 1.21	5.585 ± 0.91
Heart	0.156 ± 0.04	0.185 ± 0.02	0.278 ± 0.06	0.430 ± 0.09
Kidney	3.794 ± 0.81	3.344 ± 0.65	0.868 ± 0.12	1.110 ± 0.11
Muscle	0.111 ± 0.04	0.102 ± 0.03	0.097 ± 0.03	0.130 ± 0.05
cerebrum	0.042 ± 0.01	0.058 ± 0.03	0.081 ± 0.01	0.143 ± 0.04
cerebellum	0.032 ± 0.01	0.032 ± 0.01	0.068 ± 0.02	0.137 ± 0.04

### 3.7 PMT tolerability in healthy animals

**FIGURE 9:**
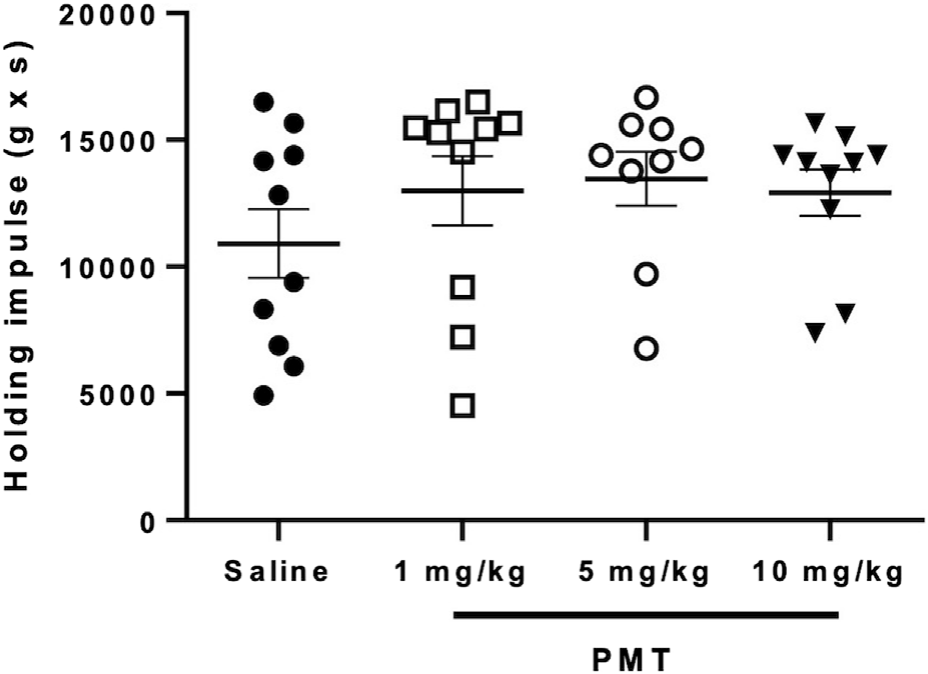
Horizontal Grid-hanging test for motor dysfunctions evaluation after the injection of saline (n = 10) or different PMT concentration (1, 5 and 10 mg/kg; n = 10 per group). Data are expressed as Mean ± SD.

The i.v. injection of increasing concentrations of PMT solution did not produce any significant effect on the behaviour of animals evaluated at 14 days with the Grid-hanging test (Figure 9). Mice treated with 1, 5 and 10 mg/kg of PMT exhibited similar muscular strength to mice injected with saline.

**FIGURE 10:**
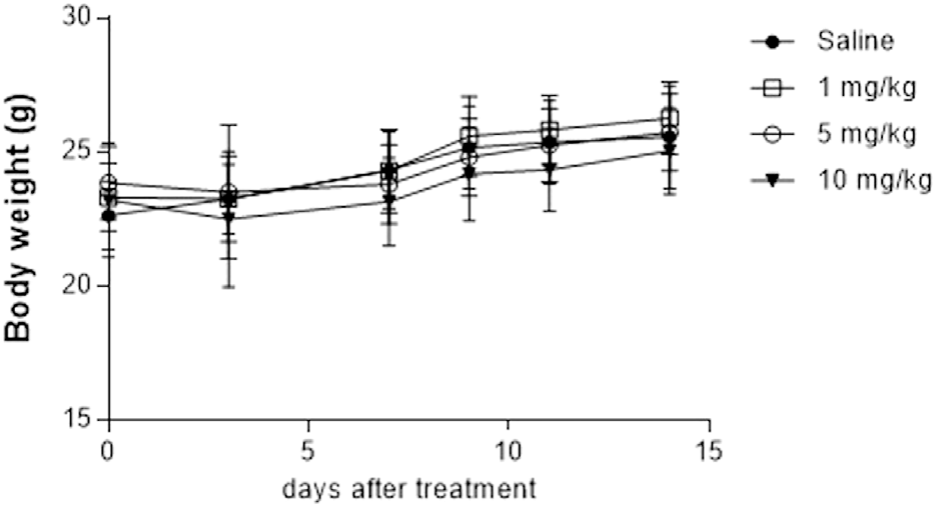
Body weight measured for the different animal groups (n = 10 each) after the injection of saline or different concentration of PMT (1, 5 and 10 mg/kg). Data are expressed as Mean ± SD.

In the days following the injection of 10 mg/kg of PMT, a subgroup of animals manifested slight distress increase, consisting in mild tachypnoea (score 1) in 5/10 mice, and body weight decrease (score 2–3) in 8/10 animals, as compared with initial values (Supplementary Figure S6). A slight decrease of body weight was observed also in animals treated with PMT 1 mg/kg (score 1 in 5/10 mice) and 5 mg/kg (score 1–2 in 5/10 mice and 4 in 1/10 mice). The spontaneous behaviour, as well as body weight, improved in the following days. In fact, no significant body mass differences were observed between animal groups at the end of the experiment (Figure 10). Moreover, the mean value of body weight between groups was not significantly different at any time point (p < 0.05, multiple comparison with one-way ANOVA).

### 3.8 Morphological analysis of selected organs

Microscopic analysis of sections stained with hematoxylin and eosin did not show any morphological change in any organs or tissues of treated animals with respect to controls (Supplementary Figure S7).

## 4 Discussion

In the past years we have developed short peptides aimed at mitigating the severe symptoms associated with mitochondrial diseases, such as MELAS and MERRF, caused by point mutations in mitochondrial tRNA genes, which currently lack effective therapies. Due to the potential susceptibility of peptide molecules to human enzymes, in this work, we have designed and evaluated three peptide-mimetic derivatives of the most active *β*32-33 peptide, named PMT, PMT-8a and PMT-8b. Since these peptide-mimetics comprise only D-amino acids, they are expected not to be substrates of human enzymes, which are specific for L a.a. and, therefore, to be more amenable to *in vivo* applications than the *β*32-33, which is entirely made of L a.a.

We demonstrated that PMT, PMT-8a and PMT-8b share important biological properties with the *β*32-33 peptide, namely,: cell membranes penetration; co-localization with mitochondria; and ability to significantly improve both viability and oxygen consumption of cells carrying either of the two point-mutations responsible for the most common and severe mt diseases, i.e., MELAS and MERRF. These results indicate that the switch from the L a. a., present in the natural *β*32-33 peptide, to the D configuration of the a. a. belonging to the PMT does not affect either membrane penetration ability or rescuing properties.

The fact that the ability to penetrate cell and mt membranes is not affected by this modification is not surprising, since this property is generally ascribed to the interactions of positively charged and hydrophobic side chains with the negatively charged heads and hydrophobic chains of membrane phospholipids, respectively. On the other hand, maintenance of therapeutic activity could not have predicted with certainty, since macromolecular interactions are usually stereospecific, with drug enantiomers often showing differences in the extent or even type of biological activity. Indeed, the *β*32-33 peptide and PMT comprise the same amino acids, in the same order along the sequence, but they have opposite side chain orientation with respect to the main chain. This implies that, if the molecular target of the *β*32-33 peptide and PMT is the same, these two molecules can have the same activity only if their target binding does not involve equally main chain and side chain atoms but is contributed predominantly by the latter. To test this hypothesis, we analysed in detail the experimentally determined bacterial structures of LeuRS/tRNA^Leu^ complexes that had been the starting point and rational basis of our Cterm and *β*32-33 peptide design (Perli et al., 2014; Perli et al., 2016). We dissected the type of intermolecular contacts established by the *β*32-33 and *β*30-31 peptides, the Cterm and the rest of LeuRS, and found that the *β*32-33 peptide region had the peculiarity of interacting with tRNA^Leu^ exclusively through side-chain atoms, whereas for all the other regions a relevant percentage of main chain contacts (i.e., between 17% and 43%) was involved. We also investigated the number of contacts involving main chain and side chain atoms in a molecular model of the human mt-LeuRS and mt-tRNA^Leu(UUR)^ complex built by AF3. While the structure of neither molecule has been experimentally determined, in recent years protein structure prediction programs based on artificial intelligence have progressed to the point of being able to produce 3D structure models with accuracy comparable to that of experimentally determined structures (Kryshtafovych et al., 2023), an achievement that was crowned by the assignment of the Nobel prize to the developers of the pioneering AlphaFold algorithm (https://www.nobelprize.org/prizes/chemistry/2024/press-release/). One of the strengths of this method is the ability to provide accurate estimates of the reliability of the model at a residue level, an ability that has been recently extended to tRNA molecules. Based on these estimate values, the large majority of the AF3 model is expected to have high or very high accuracy (88% of mt-LeuRS and 81% of mt-tRNA^Leu(UUR)^; additionally, the relative positioning of the two molecules is highly similar to that observed in known structures (as an example, compare Supplementary Figure S3; Perli et al., 2016; Figure 2). While this model cannot be expected to have the same accuracy as that of the experimental 3D structures, the fact that the mt-LeuRS region corresponding to the *β*32-33 peptide interacts with mt-tRNA^Leu(UUR)^ almost exclusively (i.e., 98% of the contacts) through side chain atoms, parallels the results of the calculations performed on experimental structures and supports the validity of the model. The ability of the β32_33 peptide region to bind the cognate tRNA through side chain atoms, exclusively in bacterial LeuRS structures, and predominantly in a human model of the complex, agrees with, and provides a possible explanation for, the highly similar therapeutic activity of the PMT and β32_33 peptide in cell models.

Based on the structural analysis, and of the conservation of the extent of the rescuing activity between the *β*32-33 peptide and the PMT, we speculate that the PMT acts with the same mechanism previously proposed for the *β*32-33 peptide, namely, by binding the target mt-tRNA^Leu(UUR)^ through its conserved side chains, and by stabilizing a native-like mt-tRNA^Leu(UUR)^ conformation able to better perform its physiological functions. As far as the interactions with mt-tRNA^Lys^ are concerned, we have previously reported that the *β*32-33 peptide is endowed with rescuing activity not only towards the cognate mt-tRNA^Leu(UUR)^ but also towards mt-tRNA^Lys^ and other mt-tRNAs, both in human cells and in the yeast model (Perli et al., 2016; Perli et al., 2020; Di Micco et al., 2014). We can speculate that the reason why the therapeutic activity of the PMT towards mt-tRNA^Lys^ is as high as that of the *β*32-33 peptide is that, as in the case of the interactions with the cognate mt-tRNA^Leu(UUR)^, the interactions of the *β*32-33 peptide with non-cognate mt-tRNAs is contributed mostly by side chains atoms. Further experiments will be required to demonstrate whether PMT directly binds mt-tRNA *in vitro* and in cell models and determines mt-tRNA stabilization, as well as to elucidate in detail the cellular mechanisms that lead to the rescuing of cellular functions.

Plasma stability experiments were then performed to assess the amenability of the investigated compounds for *in vivo* experiments. The fact that all three peptide-mimetics resulted to be remarkably more stable than the *β*32-33 peptide upon incubation in human plasma, can be explained based on the fact that PMT, PMT-8a and PMT-8b comprise only D-, and the *β*32-33 peptide only L-, amino acids; indeed, compound stabilization was the result aimed for by the replacement of L-with D-amino acids. Conversely, the decrease of PMT signal, in light of the stability of the PMT-8a and PMT-8b fragments up to 72 h, was less expected. We believe that PMT degradation by chemical, as well as biological, agents is highly unlikely, since each PMT fragment comprises a fraction of the same amino acids present in the PMT (i.e., D-amino acids 1-8 in PMT-8a and 5–12 in PMT-8b, respectively), therefore any hypothetical breakage in PMT bonds would also occur in both, or at least one of, PMT fragments. Examination of the PMT sequence highlighted that six of the eight C-terminal residues of the PMT (i.e., D-Ala 10, D-Leu 11, D-Ile 12, D-Phe 14, D-Leu 15 and D-Val 16), which are not present in PMT-8a and only three of which are present in PMT-8b, have a strong hydrophobic character and, therefore, might induce PMT aggregation and/or sequestration by plasma proteins endowed with hydrophobic pockets, such as serum albumin.

Since PMT had highest rescuing activity on both cell mutants and was >25% detectable after 72 h of incubation in human plasma, it was selected for further studies.

First, we demonstrated that PMT is neither cytotoxic nor mitotoxic for m.3243A>G mutant cybrids at concentrations up to 20 μM, for m.8344A>G mutant cybrids at concentrations up to at least 50 μM, and for healthy control cells at concentrations up to 40 µM.

Then, we showed that PMT is well tolerated by wild-type mice after a single i. v. acute administration of 1, 5 and 10 mg/kg (this last value corresponding to 0.3 mg for a 30 g mouse), as indicated by the lack of permanent adverse effects on behaviour and body weight during the 14 days of observation period and necroscopic post-mortem evaluation.

Finally, we showed that the PMT administered to healthy mice by i.v. injection localizes at the level of all tissues, including those that are most affected by pathological point mutations in mt-tRNA, namely, heart, skeletal muscle and even brain. PMT radiolabelling with ^64^Cu allowed us to follow the *in vivo* distribution of the peptide in the body districts of healthy animals over time. Tissue-to-blood ratio values of radioactivity concentration showed clearance over time in liver, kidneys and spleen, and substantially stable levels of ^64^Cu-PMT in the majority of tissues including the brain, sustained by blood recirculation of ^64^Cu-PMT. Importantly, the kinetic profile of ^64^Cu-PMT in the brain differs from that previously reported for free ^64^Cu distribution in healthy animals (Peng et al., 2018). In those studies, radioactivity concentration of free ^64^Cu immediately reached 0.4–0.6 %ID/mL in different cortical regions and remained stable up to 24 h after injection. Conversely, ^64^Cu-PMT concentration in the brain is initially very low (i.e., 0.026 %ID/g after 1 h) and slowly increases over time (i.e., 0.043, 0.168 and 0.502 %ID/g after 3, 24 and 48 h). This observation, together with the stability of ^64^Cu-PMT in solution at room temperature for 24 h, and the fact that choroid plexus signal, which is the main exchange system for free copper in the brain, was not present in the PET images, indicate that most of the radioactivity signal measured in animals was likely to be generated by ^64^Cu-PMT distribution and that, although a small amount of free copper could be present, its contribution to the radioactivity signal is negligible.

The fact that the PMT is able, by itself, to cross the blood-brain barrier is particularly striking, since this anatomical-functional structure is designed to be penetrable by only a few selected substances and, therefore, it is a well-known obstacle to the passage of many therapeutics. Indeed, about 0.5% and 1% of PMT i. v. injected in animals for imaging purposes was localized in cerebrum and cerebellum after 48 h, respectively (see Table 3).

Further experiments will have to be performed to identify the optimal administration protocol to reach a PMT concentrations in target organs of healthy mice comparable to that active in cell models (i.e., 5 µM), in both acute and chronic evaluation experiments, using different administration routes and, if required, implementing delivery strategies. As an example, while *in vivo* biodistribution data indicates that the PMT is able to pass the BBB by itself, PMT concentration in the brain at each time interval was lower than in other tissues; therefore, strategies to increase brain localization should be implemented. Intranasal administration offers a direct route to the brain, which does not involve BBB penetration, and might be explored by administering the PMT as such. Additionally, the PMT may be joined to, or encapsulated within, nanoparticle-based carriers of different chemical nature, some of which have been developed with the contribution of members of our research groups (Falvo et al., 2016; Falvo et al., 2021; Iannone et al., 2025). A particularly attractive nanoparticle carrier, in our opinion, is represented by the heavy chain of human ferritin (HFt), a highly soluble, non-immunogenic, biodegradable protein that can be easily obtained with high yields, low cost and high purity able, passes the BBB via receptor-mediated transcytosis, and has been successfully used to deliver drugs to the brain following both intravenous and intranasal administration (Marrocco et al., 2024, and references therein).

Knowledge of the optimal PMT administration protocol gained by preclinical studies will be exploited to choose the starting administration dose in putative future clinical studies, taking into account the well-known impact of the metabolic differences among species on drug pharmacokinetics (Nair and Jacob, 2016).

## Data Availability

The original contributions presented in the study are included in the article/Supplementary Material, further inquiries can be directed to the corresponding authors.
