# Supplementary material for: Peptide-mimetics derived from leucyl-tRNA synthetase are potential agents for the therapy of mt-tRNA related diseases
Source: Front Pharmacol. 2025 Jul 28;16:1607343. doi: 10.3389/fphar.2025.1607343 (PMC12336229; doi:10.3389/fphar.2025.1607343)
Supplement: Supplementary file 1 [file DataSheet1.pdf]

Supplementary Material

1. Supplementary Tables

Supplementary Table 1. Animal monitoring grid. Summary of parameters and associated scores of animal welfare included in the periodic observation.

PARAMETER	DESCRIPTION	SCORE
Body weight loss	0-5 %	1
	5-10%	2
	11-15%	3
	16-20%	4
	>20%	5
Coat condition	Slightly dishevelled	1
	Shaggy and dirty, moderate piloerection	2
	Dishevelled, lack of piloerection	3
Dehydration	Without skin fold relief	1
	With skin fold relief	3
Breathing	Tachypnoea	1
	Dyspnoea	3
Spontaneous behaviour	Normal locomotion	0
	Reluctance in locomotion, slightly abnormal gait	2
	Lethargy, apathy, notable gait abnormality	3
	Significant movement problems	4
	Immobility for more than 12 hours	5
Reaction to handling	Normal curiosity, attentive and alert	0
	Tense and nervous about handling	1
	Weak to grip	2
	Marked distress on manipulation (tremors, vocalization, and aggressiveness)	3

SCORE	INTERVENTION
1	Review monitoring frequency
2-3	Evaluate the possibility to administrate additional care (e.g., extra fluids)
4	Additional care administration in agreement with the veterinarian
5	Severe suffering, practice euthanasia

Supplementary Material

2. Supplementary Figures

Supplementary Figure 1

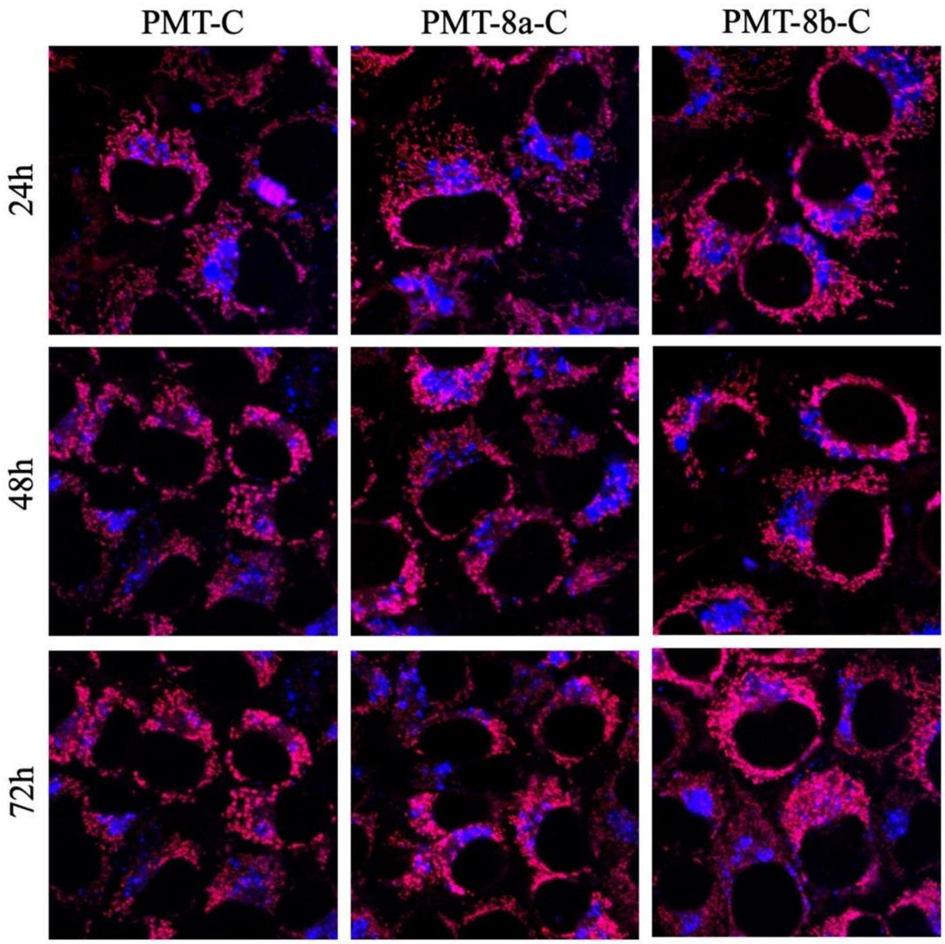

Constructs	PMT-C			PMT-8a-C			PMT-8b-C		
Time	24h	48h	72h	24h	48h	72h	24h	48h	72h
PCC	0.79±0.02	0.76±0.01	0.78±0.03	0.8±0.02	0.79±0.01	0.78±0.03	0.8±0.01	0.77±0.03	0.82±0.02

PMT, PMT-8a and PMT-8b penetrate cell membranes and co-localize with mitochondria up to 72 hours.
Upon exogenous administration to mutant cells, PMT and PMT fragments (PMT-8a and PMT-8b) penetrate cell membranes and colocalize with mitochondria up to 72 hours. Cybrids were incubated with 0.25 μM of constructs and imaged after 24-, 48- and 72 hours. Half an hour before imaging, cells were stained with Mitotracker Red. PCC: Pearson's Correlation Coefficient (mean±SEM of at least three images). Names of all constructs are followed by "-C" to indicate that they are covalently linked to the Cy5 fluorescent dye.

Supplementary Figure 2

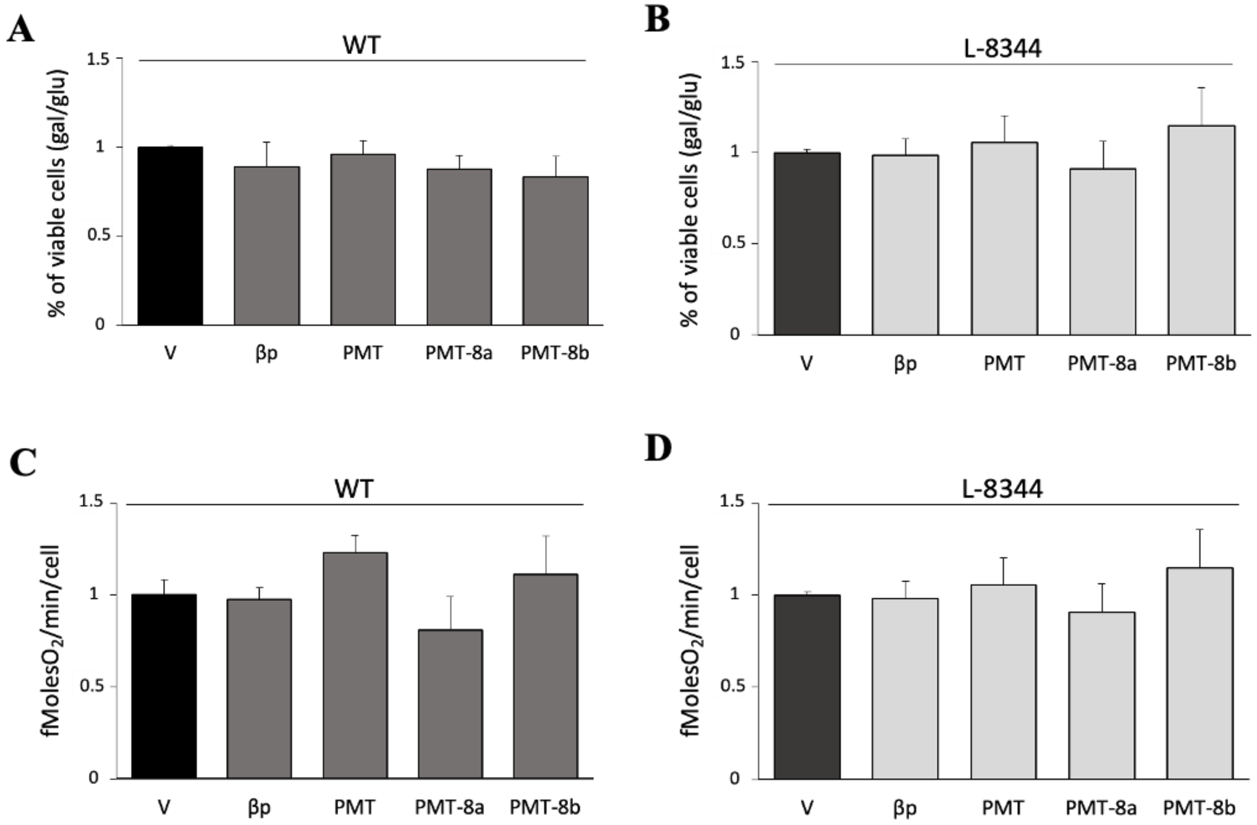

PMT and PMT fragments do not affect viability or oxygen consumption of control cells.
(A-B) Viability of treated WT (**A**) and L-8344A>G (**B**) cells evaluated after 24 h incubation in galactose medium. The number of viable cells in galactose is normalized to the number of viable cells in glucose at the same time point.
(C-D) Rate of oxygen consumption of treated WT (**C**) and L-8344A>G (**D**) cybrids, evaluated after 36 h. Data are expressed as treated/vehicle ratio and are mean±SEM of the last three independent experiments.
V: vehicle; WT: wild type; L-8344: cells with extremely low levels of mutation m.8344A>G in mt-tRNA^{Lys}

Supplementary Material

Supplementary Figure 3

A

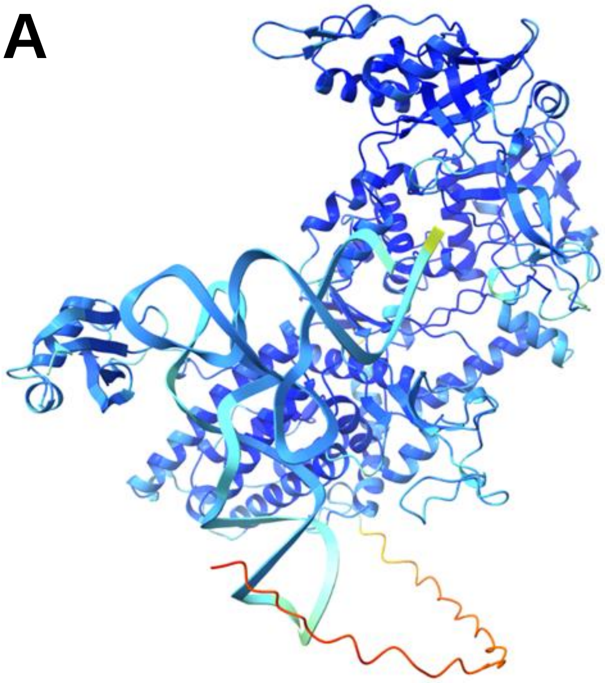

B

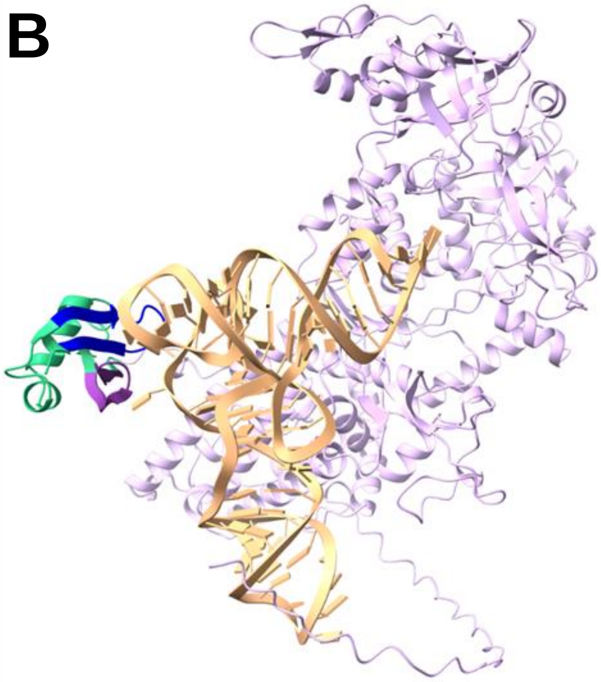

C

				264	271	669	674	683	687	690	693	750	751	754	757	761	765	842	847	849	852	889	891	893	894	896	898	900
		A		G	H	A	K	D	G	R	Q	N	S	S	M	N	Q	A	N	A	K	F	S	R	T	L	N	L
	B			T	H	S	S	T	H	H	H	H	H	H	H	H	T	E	T	E	E	E		S	S	E	E	E
				4	7	2	6	9	2	9	4	2	3	3	2	3	4	1	6	3	1	6	2	9	2	5	1	2
3	U	*	4		1.0																							
4	A	*	6	1.0																								
5	A	*	3	0.1																								
11	C	*	0					1.0																				
12	A	*	4			1.0		2.0																				
13	G	*	8									2.0																
14	A	*	7				1.0						1.0															
18	C	*	3																					8.4				
19	G	*	2																					4.0				
20	G	*	9																1.0				1.3		2.0	0.3	1.0	
21	U	*	9															0.1		0.2	1.1							
25	C	*	8											1.0		1.0												
26	G	*	9					5.2	1.0						0.2	2.0												
27	C	*	9					1.2	0.1	4.0																		
28	A	*	3							4.0																		
40	A	*	4							1.0	3.0																	
41	C	*	8							2.0	0.1																	
43	U	*	4														4.0											
58	C	*	9																3.1			0.9					3.0	0.2
59	A	*	1																2.1									

Interactions between human mt-LeuRS and mt-tRNA$^{Leu(UUR)}$ in the molecular model of the complex built by AlphaFold3.

(A) Molecules are shown as ribbons and coloured by AF3 estimated accuracy expressed by pLDDT (*i.e.*, predicted local distance difference test) values: blue, cyan, yellow and orange indicate very high (pLDDT ≥ 90), high (70 ≤ pLDDT < 90), low (50 ≤ pLDDT < 90) and very low (pLDDT < 50) confidence in the prediction, respectively (Abramson et al, 2024).

(B) Molecules are shown as ribbons and coloured as follows: blue, β32_33 peptide; magenta, β30_31 peptide; green, human mt-LeuRS Cterm domain except the β32_33 and β30_31 peptide regions; lilac, human mt-LeuRS except the Cterm domain; orange, human mt-tRNA$^{Leu(UUR)}$.

(C) Matrix of contacts between the two molecules calculated by FACE2FACE (Di Micco et al, *under revision*). Chain A: mt-LeuRS. Chain B: mt-tRNA$^{Leu(UUR)}$. The 1st, 2nd, 3rd and 4th row and column of the matrix report the name, number, secondary structure (only for chain A) and solvent-accessible surface area (SASA) buried at the interface of each amino acid and nucleotide residue, respectively. Cell colour indicates the type of atoms involved in the contacts between each amino acid and each nucleotide (*i.e.*, only polar: blue; only non-polar: yellow; both polar and non-polar: green). The colour shade is darker and lighter for higher and lower numbers of contacts, respectively. The number of polar and non-polar contacts between residues are shown as integers within these cells, and are followed and preceded by a dot, respectively.

References

Abramson, J., Abramson, J., Adler, J., Dunger, J., Evans, R., Green, T., et al. (2024) Accurate structure prediction of biomolecular interactions with AlphaFold 3. Nature. 630:493-500. 10.1038/s41586-024-07487-w.

Di Micco P, Incarnato M, Pascarella G, Via A, Morea V. Fast, comprehensive and user customizable macromolecule interface analysis with FACE2FACE. J. Chem. Inf. Model., *under revision*.

Supplementary Material

Supplementary Figure 4

A

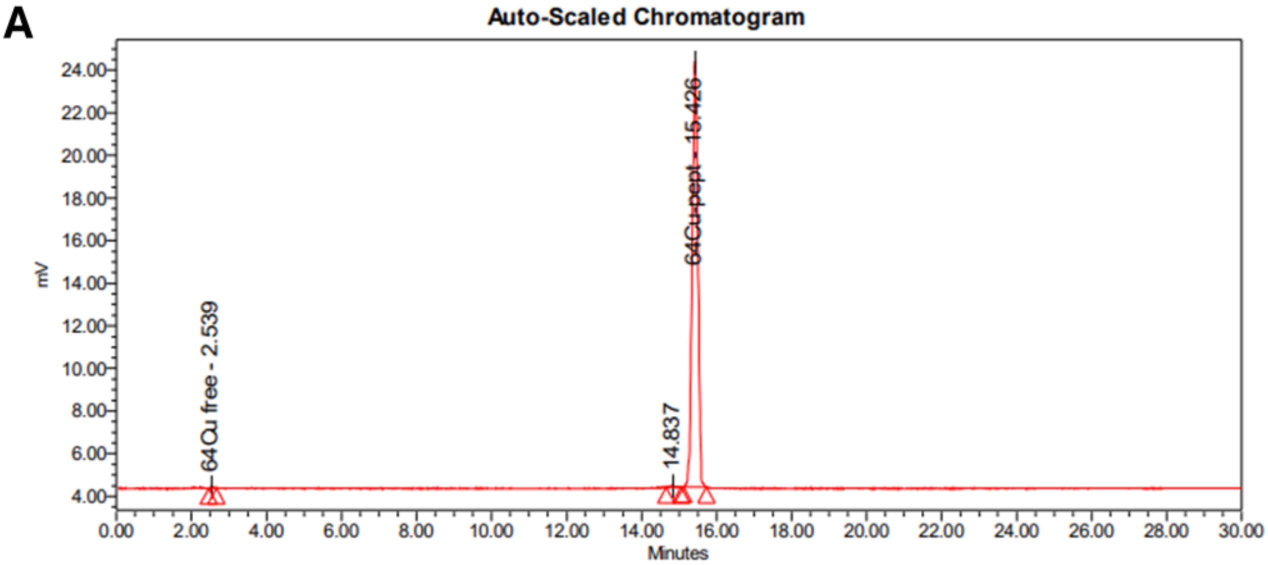

B

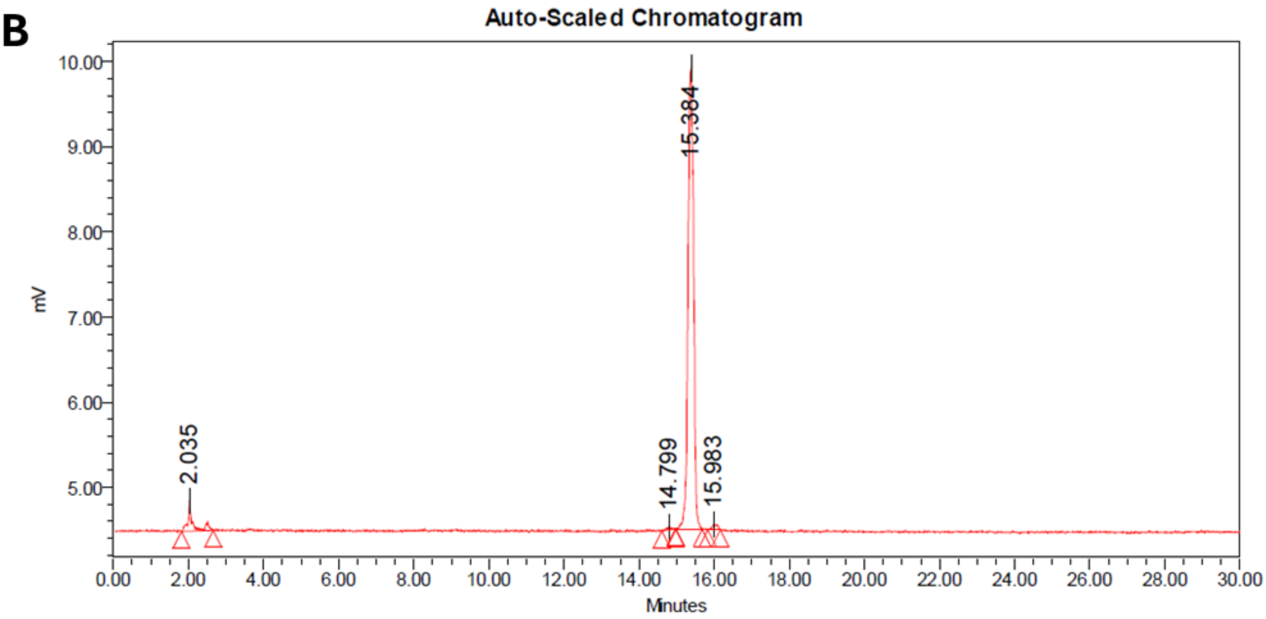

	Name	RT	Area	Height	% Area
1	Free ^{64}Cu-Cu^{++}	2.035	3013	354	5.01
2	Unknown	14.799	263	29	0.44
3	^{64}Cu-PMT	15.384	56212	5475	93.46
4	Unknown	15.983	658	61	1.09

Radiochromatogram of ^{64}Cu-PMT.

Radio-HPLC chromatogram of ^{64}Cu-PMT at the end of the radiosynthesis **(A)** and after 24 hours in saline solution at room temperature (RT) with relative peaks quantification **(B)**.

Supplementary Figure 5

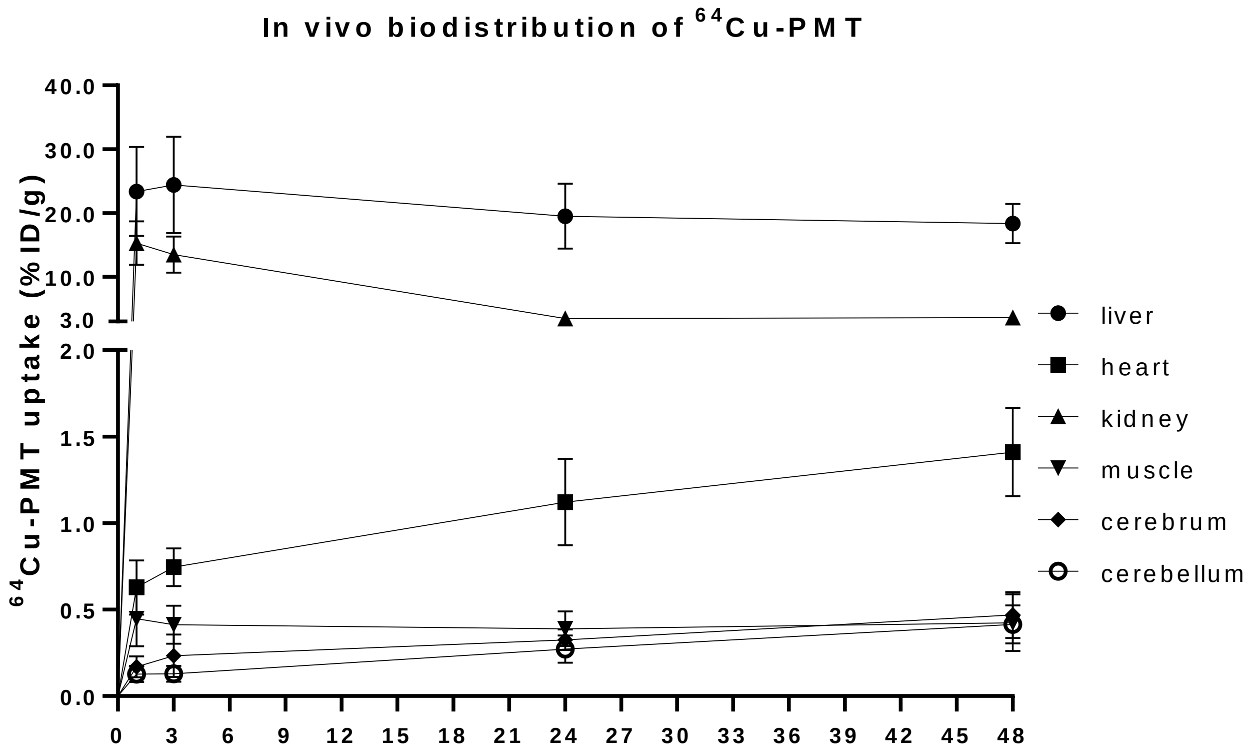

Kinetics of *in vivo* biodistribution of ^{64}Cu-PMT in brain and selected peripheral tissues of WT mice.

Animals were injected i.v. with ^{64}Cu-PMT and acquired by CT-PET at different time points from injection. Data are expressed as percentage of injected dose per gram of tissue (%ID/g) and are mean±SD of 5 animals per time point.

Supplementary Figure 6

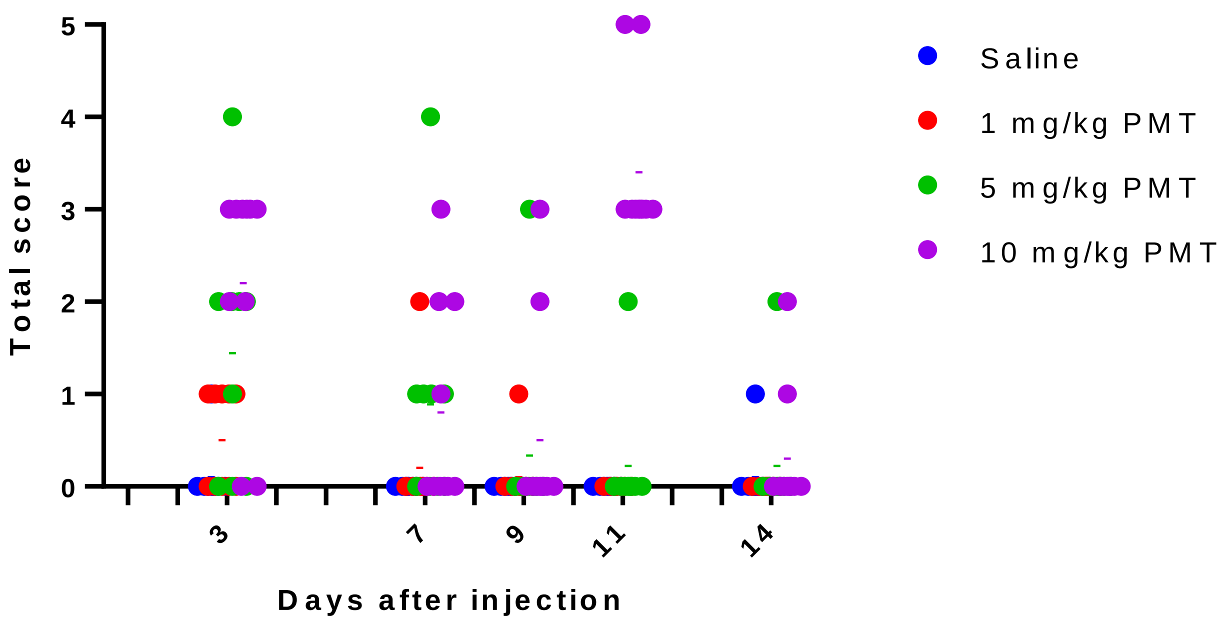

Tolerability monitoring score.

Graphical representation of score evaluation after the injection of saline (n=10) or different PMT concentration (1, 5 and 10 mg/kg; n=10 per group) in wild type animals.

Supplementary Figure 7

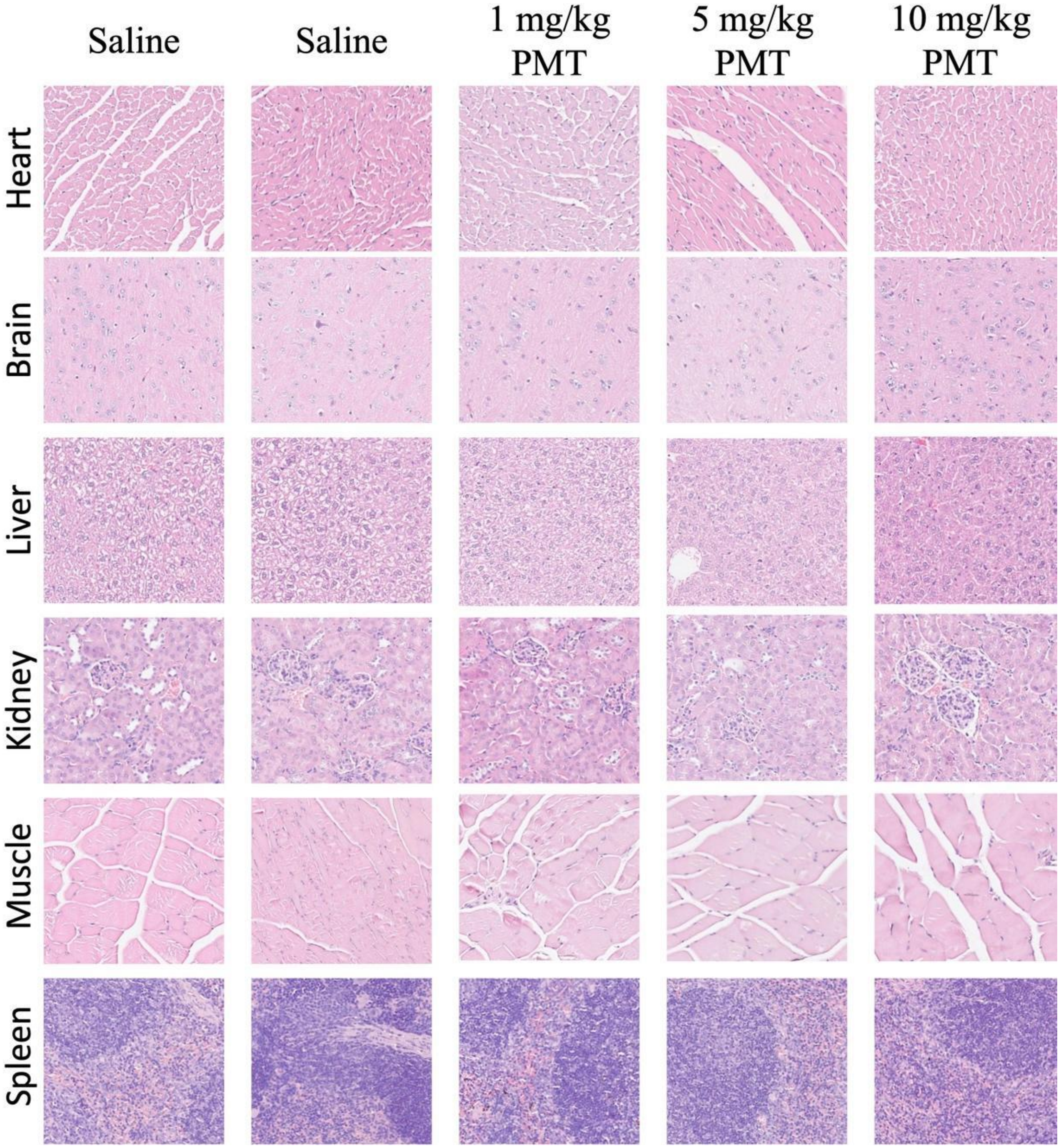

Microscopic analysis of sections stained with haematoxylin and eosin did not show any morphological change in any organs or tissue.

Haematoxylin-eosin of mice’s heart, brain, liver, kidney, muscle, and spleen treated with different concentrations of PMT (1, 5 and 10 mg/kg). Histological analysis did not reveal any morphological change in any organs of PMT treated mice with respect to controls. Original magnification: 10X.
